# Impact of Salinity on Cell Surface Chemistry of Cyanobacteria From Freshwater, Marine and Alkaline Environments: A Hidden Phosphorus Engine

**DOI:** 10.1111/1462-2920.70180

**Published:** 2025-09-30

**Authors:** David Aceituno‐Caicedo, Nigarsan Kokilathasan, Yuwei Zhao, Maria Dittrich

**Affiliations:** ^1^ Biogeochemistry Group, Department of Physical and Environmental Sciences University of Toronto Toronto Canada; ^2^ Earth Sciences Department University of Toronto, University of Toronto Toronto Canada

**Keywords:** cell surfaces properties, cyanobacteria, Fourier transform infrared spectroscopy, functional surface groups, salinity, *Spirulina* sp., *Synechococcus* sp., *Synechocystis* sp., x‐ray photoelectron spectroscopy

## Abstract

Climate change is altering ocean salinity, impacting cyanobacteria, key primary producers with vital ecological roles. While cyanobacterial adaptations to salinity are well studied, molecular cell surface chemistry changes remain underexplored. This study examines the impact of salinity on surface properties of freshwater *Synechocystis* sp. PCC6803, marine *Synechococcus* sp. PCC8806 and alkaliphilic *Spirulina platensis*. Species were cultured under salinities of 2‰, 6‰, 10‰, 30‰ and 60‰. Surface chemical composition and content were characterised using infrared and x‐ray photoelectron spectroscopy and potentiometric titration. All strains exhibited salinity‐dependent changes in surface charge and functional group expression, reflecting distinct adaptation strategies. In non‐marine strains, salinity stress led to decreased phosphoryl signals and increased lipid saturation, consistent with phospholipid replacement and reduced membrane fluidity. We propose membrane phospholipids as a phosphorus reservoir, mobilised to support biosynthesis and ion homeostasis. In contrast, *Syn*. PCC8806 increased phosphoryl‐associated signals and membrane fluidity across salinity conditions, consistent with phosphatidylglycerol enrichment to maintain photosynthetic function. Surface chemical shifts support a model where membrane remodelling is central to species‐specific acclimatisation, balancing nutrient conservation with functional integrity. This work enhances understanding of microbial adaptation under osmotic stress and provides insight for predicting cyanobacterial blooms, designing biotechnological systems.

## Graphical abstract

The study examines the impact of salinity on surface properties of freshwater *Synechocystis* sp. PCC6803, marine *Synechococcus* sp. PCC8806 and alkaliphilic *Spirulina platensis.* The differences in membrane restructuring between marine and non‐marine species highlight phosphorus as an important energy resource during membrane restructuring under high salinity.

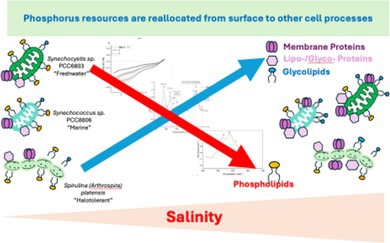

## 1 Introduction

Anthropogenically triggered climate change is accelerating the global water cycle (Cheng et al. 2020), amplifying ocean salinisation patterns (Pierce et al. 2012). As a result, marine coastal regions (Durack et al. 2012) and some freshwater regions (Cañedo‐Argüelles et al. 2013) are increasing in salinity. Salinity shifts impose new ecological pressures on aquatic organisms (Schaum et al. 2016), forcing physiological adaptations to fluctuating environmental conditions (Arromrak et al. 2022). Changing salinity is particularly consequential for microorganisms like cyanobacteria that play key roles in biogeochemical cycling in aquatic ecosystems (Singh et al. 2020). An understanding of the consequences, adaptation and management requires studying cyanobacterial cell surface response to elevated and fluctuating salinity (Singh et al. 2020).

Under excessive salinity conditions, cyanobacteria experience osmotic stress (Allakhverdiev et al. 2000), water loss, reactive oxygen species (ROS) production (Singh et al. 2022), decreased photosynthetic activity (Allakhverdiev et al. 1999, 2001) and altered membrane fluidity and permeability (Singh et al. 2022). Many cyanobacteria taxa demonstrate impressive halotolerance and salt‐stress response mechanisms (Liang et al. 2020), including osmo‐protectant and protective protein synthesis (Pade and Hagemann 2014), enhanced ion transport, production of heterogeneous exopolysaccharide substances (EPS) (Qiu et al. 2012) and restructuring of cell envelopes and membranes to modify membrane fluidity (Joset et al. 1996). Some taxa, like *Synechococcus*, modulate biomass production enriched with pigments, proteins, carbohydrates and lipids in response to salinity changes (Rosales et al. 2005).

The energetically expensive response to ionic and osmotic stress depends on the nature and degree of stress (Joset et al. 1996) and requires a highly regulated response (Klähn et al. 2021). Various osmo‐adaptive cellular mechanisms (Wood 2015) and biosynthesis of compatible solutes (Pade and Hagemann 2014) have been defined in the literature. For example, *Spirulina platensis* lowers photosynthetic activity and increases respiration to supply energy for sodium ion extrusion (Vonshak 1997) and glucosylglycerol (GG) and trehalose production (Warr et al. 1985) and it also increases in absolute carbohydrate content (Vonshak et al. 1988). Furthermore, the cyanobacterial salt‐stress responses at the transcriptomic (Wang et al. 2013), proteomic (Pandhal et al. 2008) and metabolomic levels have highlighted that cellular responses to salt‐stress are species‐specific.

The cyanobacterial cell envelope includes an outer membrane (OM), peptidoglycan layer (PD) and inner membrane (IM) (Silhavy et al. 2010) and in some species, like *Synechococcus* sp., a surface layer (S‐layer) composed of identical glycoproteins (Schultze‐Lam and Beveridge 1994). Previous studies demonstrate changes in cell envelope composition under salinity changes (Huflejt et al. 1990), which impact surface charge due to changes in charged functional groups (Khomutov et al. 1990). These functional groups include phosphoryl, carboxyl, hydroxyl among others (Cox et al. 1999) and their abundance can vary depending on the cells' environment (Liu et al. 2016). Phosphorus in surface phosphoryl groups is a key metabolic currency that cells may reallocate to metabolic purposes under phosphorus starvation (Hiyoshi et al. 2024). Salinity‐driven turnover of phosphorus‐containing membrane groups could then possibly alter surface charge and free phosphorus for ATP generation (Andersson et al. 2003, 2005). The cell surfaces are crucial for nutrient interaction (Fiebig et al. 2014) and interactions with dissolved organic matter (Kujawinski 2011), metals (Yee et al. 2004) and pollutants (Vijayaraghavan and Yun 2008). The changes in surface properties provide valuable insight into surface cell–cell interactions as well as cells' behaviour in changing environments (Custódio and Mano 2016). However, how salinity adaptations may impact the chemical molecular surface properties of marine and freshwater cyanobacteria specifically is understudied.

Despite extensive research into transcriptomic and physiological responses to salt stress, comparatively little is known about how cyanobacteria alter their surface‐level chemical architecture during salinity acclimatisation, including functional group composition, membrane lipid structure and elemental distributions. Surface properties are crucial for understanding interactions with nutrients, ions and other cells, yet the molecular‐level mechanisms governing the surface adaptations remain unclear. In this study, we address the following main questions: (1) How does salinity exposure alter the surface chemistry of cyanobacteria across different ecological origins, including freshwater, marine and alkaline? (2) What are the functional group changes and membrane reorganisation patterns that reflect adaptive strategies or species‐specific responses? (3) How do surface‐level changes in functional groups and phosphorus content remodel the membrane as part of a metabolic adaptation to salinity stress? We further explore whether this potential reallocation of surface phosphorus could serve as a regulatory mechanism for energy balance, a concept we refer to as a ‘phosphorus engine’. Consequently, understanding the changes in surface properties of salt‐acclimatised cells may help to better predict environmental interactions and extrapolate impacts on ecological, biogeochemical and biotechnological applications.

To investigate these questions, we focus on three representative cyanobacteria strains that inhabit freshwater, marine and alkaline environments, respectively. *Synechocystis* sp. PCC6803 (*Syn*. PCC6803) is a freshwater strain and is a model gram‐negative cyanobacteria species that has been characterised for its overall proteomic response to salt stress (Fulda et al. 2006), proteomic membrane response to salt stress (Huang et al. 2006), ion transport mechanisms (Tsunekawa et al. 2009) and transcriptomic acclimatisation to high or fluctuating salt conditions (Klähn et al. 2021). *Synechococcus* sp. PCC8806 (*Syn*. PCC8806) is a marine strain that has been studied for cell surface biomineralisation potential (Liang et al. 2013) and adaptations to varying media conditions (Paulo et al. 2018). However, *Syn*. PCC8806 has not yet been classified into a specific clade for its ecotype (Schoch et al. 2020). The genus *Synechococcus* at large has been studied for photosynthetic response to salt stress (Allakhverdiev et al. 2000), compatible solute synthesis (Liang et al. 2020) and biochemical responses to salt stress in general (Singh et al. 2022). *S. platensis* , also known as *Arthrospira platensis*, has been studied for its production of compatible solutes (Warr et al. 1985), changes in metabolism (Vonshak et al. 1988), growth rate and ion accumulation (Kebede 1997), biomass production and biochemical composition (Markou et al. 2023). *S. platensis* has been observed to have high salt tolerance though can be found in high abundance under freshwater conditions.

In the present study, we exposed selected strains to different salinity levels compared to their natural environments and compared the surface properties. We characterised the molecular cell surfaces by applying attenuated total reflection fourier transform infrared (ATR‐FTIR) spectroscopy to identify the functional groups and estimate the proportion of lipid saturation in surface lipids. x‐Ray photoelectron spectroscopy (XPS) was applied to quantify the overall content of the surface functional groups and bonds. Potentiometric titration (PT) with linear programme modelling (LPM) was applied to model changes in surface charge due to adjustments in functional group content across pH.

## 2 Methods

### 2.1 Cyanobacterial Species and Culture Conditions

Three species, *Synechocystis* sp. PCC6803 (P68), *Synechococcus* sp. PCC8806 (P88) and *Arthrospira* (*Spirulina*) *platensis* (SP), were received from the Pasteur Culture Collection. P68, P88 and SP were cultivated in BG11, ASN‐III and Zarrouk's media, respectively, and maintained in stock batch cultures in 1 L Erlenmeyer flasks. By adding or reducing NaCl in media, we modified final salinity concentrations and grew cultures under different experimental conditions. These conditions are labelled as freshwater (F), saline (S) and hypersaline (H). Final salinity concentrations for BG11 cultures were 2‰ for the regular medium (P68‐F) and 30‰ for the salinity treatment (P68‐S). Salinity concentrations for ASN‐III were 30‰ (P88‐S) for the regular medium, 60‰ (P88‐H) for the hypersaline treatment and 6‰ (P88‐F) for the freshwater treatment. Salinity concentrations for Zarrouk's media were 10‰ (SP‐F) for the regular medium, 30‰ (SP‐S) for the saline treatment and 60‰ for the hypersaline treatment. P concentrations in BG11, ASN‐III and Zarrouk's media were approximately 0.2, 3.3 and 4.4 mM, respectively, from K_2_HPO_4_·3H_2_O. All cultures were grown under aerobic conditions with a constant white light source of 4320 lm. *S. platensis* was grown at approximately 30°C, whereas *Syn*. PCC6803 and *Syn*. PCC8806 were grown at approximately 20°C. For the surface property characterisation, we harvested cells at the stationary growth phase. Details of the cultures are in Figure S1 and Table S1).

Cells were harvested by centrifuging at 4200 rpm for 10 min at 20°C using a Thermo Fisher Scientific Centrifuge Sorval ST 16R. The medium was decanted and cells were resuspended and washed three times in 18.2 Ω milli‐Q water. After the final wash, the pelleted cells were frozen at −80°C and freeze‐dried using a FreeZone 2.5 L Benchtop Freeze Dryer at −48°C and 0.12 mbar until a stable weight was obtained. Freeze‐dried cells were stored at −80°C until use. Replicate analyses were conducted using two independent biological cultures per treatment for each strain.

### 2.2 PT

Surface site concentrations were determined using duplicate dynamic endpoint acid–base titrations of the cyanobacterial suspensions in the background 0.1 M NaNO_3_ electrolyte solution. 0.1 M NaOH and 0.1 M HCl solutions were used for acid–base titrations and were prepared with 18.2 Ω Milli‐Q Water. Titrant solutions were degassed with N_2_ gas for 30 min prior to titrations to dissipate O_2_ and CO_2_. NaOH concentrations were confirmed through the titration of 3 mL aliquots with standard 0.1 M HCl and were used to confirm the HCl titrant concentration. Background electrolyte 0.1 M NaNO_3_ solutions were prepared to resuspend cells at a concentration of 3 mg dry wt/mL. The surface binding sites were calculated based on charge excess and the functional group determination was performed using a LPM based on previous works (Cox et al. 1999; Dittrich and Sibler 2005). Details of titration methods are in the Supporting Information.

### 2.3 ATR‐FTIR Spectroscopy

The cyanobacterial samples were analysed in duplicate using a Bruker Tensor II ATR spectrometer. All measurements were performed at room temperature by the accumulation of 64 spectra collected from 4000 to 400 cm^−1^ at a spectral resolution of 1 cm^−1^. The crystal was cleaned with pure isopropanol between measurements. The background spectrum was recorded in the same conditions without a sample on the crystal. The spectra were normalised to the major amide I peak (~1646 cm^−1^) using OriginPro 2024 software. Peak areas of methyl (*n*‐CH_3_) and methylene (*n*‐CH_2_) peaks in the hydrocarbon range (3100–2800 cm^−1^) were used to calculate the aliphatic CH_3_/CH_2_ absorbance ratio (*R*_3/2_) to estimate the degree of branching and chain length of the aliphatic hydrocarbons in the cell sample (Igisu et al. 2009). Beam penetration depths can range from 0.5 to 2 μm, though Dittrich and Sibler (2006) and Jiang et al. (2004) demonstrate that ATR‐FTIR of cells mostly reflect properties of the cell wall and not whole‐cell chemical composition. Detailed fingerprint, high‐frequency and lipid deconvolution spectra can be found in Figures S1–S14.

### 2.4 XPS

XPS spectra were acquired in duplicate using a Thermo Scientific K‐Alpha photoelectron spectrometer with monochromatized Al Kα X‐ray radiation. The major elements carbon, oxygen and nitrogen were recorded as C1s, O1s and N1s, respectively. Individual peak and survey analyses were obtained for each sample with a pass energy of 200 and 1 eV step. High‐resolution spectra of C1s, O1s and N1s were measured using a pass energy of 150 and 0.1 eV step. To fix the C1s component to C–C bonds, 284.8 eV was set as the binding energy scale. Avantage software was used to fit the XPS spectra peaks. The atomic concentration ratios were calculated based on the fitting of the peak areas normalised to the basis of the acquisition parameters and sensitivity factors provided by the manufacturer. XPS penetrates a few nanometres into the surfaces, allowing for the detection of surface chemical composition (Paulo et al. 2018). Details of the weight fraction ratio calculations are in the Supporting Information.

### 2.5 Data Analysis and Statistics

Spectra plotting and peak deconvolution of ATR‐FTIR data were performed using OriginPro 2024. Principal component analysis (PCA) was also conducted in OriginPro 2024 on monoprotic ligand site concentrations derived from LPM of PT data, across the pH range for each treatment and species (Figures S3–S5 and Tables S2–S7). PCA was used to identify covariation patterns in functional group abundance and pKa values. Clustering in PCA space reflects shifts in functional group abundance at specific pKa ranges, which identifies which groups and acidity ranges changed the most based on the treatment.

## 3 Results

To address our three research questions, (1) how salinity alters the surface chemistry of cyanobacteria from different environments, (2) whether patterns are species‐specific or conserved and (3) whether surface phosphorus depletion suggests a metabolic reallocation mechanism, we present results by species (Sections 3.1, 3.3). Each species' response to salinity stress is assessed using PT with LPM, ATR‐FTIR and XPS.

### 3.1 Surface Chemical Responses of Syn. PCC6803 (P68)

#### 3.1.1 Surface Site Modelling

**FIGURE 1:**
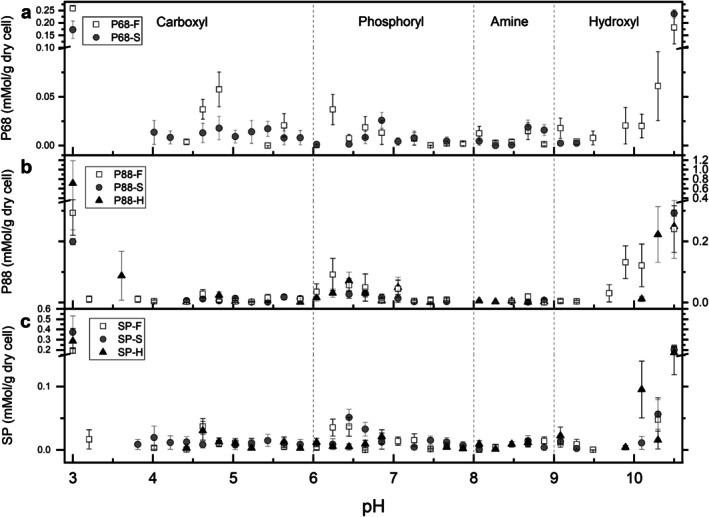
Surface site concentration (*y* axis) and distribution (*x* axis) from potentiometric titration modelling across salinity treatments. Surface functional group concentrations (mol/g) were modelled using the linear programming method (LPM) for (a) *Synechocystis* sp. PCC6803 (P68), (b) *Synechococcus* sp. PCC8806 (P88) and (c) *Spirulina platensis* (SP) across freshwater (–F), saline (–S) and hypersaline (–H) growth conditions. Functional groups include carboxyl, phosphoryl, hydroxyl (acidic) and amine (basic). Note that the species abbreviations (P68, P88, SP) and salinity levels (–F, –S, –H) are consistently applied across figures.

PT coupled with LPM revealed distinct shifts in surface functional group composition of *Syn*. PCC6803 following salt acclimatisation. The P68‐S treatment demonstrated an overall reduction in the total concentration of negatively charged carboxyl, phosphoryl and hydroxyl groups and a relative increase in positively charged amine groups compared to the P68‐F cultures (Figure 1a, Table 1). Modelling of the functional group distribution using pKa values showed that carboxyl groups in P68‐S were more narrowly distributed between pH 4.0 and 5.8, whereas P68‐F exhibited a broader distribution across the acidic range (Figure 1a). Hydroxyl groups in P68‐S were shifted towards higher pKa values (~10.5), showing altered protonation behaviour or changes in biomolecule composition.

**TABLE 1:** Functional group ligand concentrations (sum total and percent change) determined from titration experiments by means of the LPM.

Functional group (mMol/g dry cell)	pKa	Total functional group concentration, mMol/g dry cell (% change)							
		P68‐F^a^	P68‐S	P88‐F	P88‐S^a^	P88‐H	SP‐F^a^	SP‐S	SP‐H
Carboxyl	3–5.8	3.77E−01	2.82E−01 (−25.2%)	3.95E−01 (+45.8%)	2.71E−01	8.33E−01 (+207.4%)	2.87E01	4.86E−01 (+69.3%)	3.61E−01 (+25.8%)
Phosphoryl	6–8	8.80E−02	5.35E−02 (−39.2%)	3.08E−1 (+224.9%)	9.48E−02	2.03E−01 (+114.1%)	1.31E−01	1.44E−01 (+9.9%)	5.82E−02 (−55.6%)
Amine	8–9	3.53E−02	3.97E−02 (+15.9%)	2.42E−02 (+84.7%)	1.31E−02	1.82E−02 (+38.9%)	2.94E−02	1.90E−02 (−35.4%)	3.02E−02 (+2.7%)
Amine (hydroxyl)	9–10	3.12E−01	2.41E−01 (−22.8%)	5.33E−01 (+82.5%)	2.92E−01	4.82E−01 (+65.1%)	2.74E−01	2.83E−01 (+3.3%)	3.11E−01 (+13.5%)

^a^ Percent changes are compared to base medium conditions, which are freshwater for P68 and SP and saline for P88.

**FIGURE 2:**
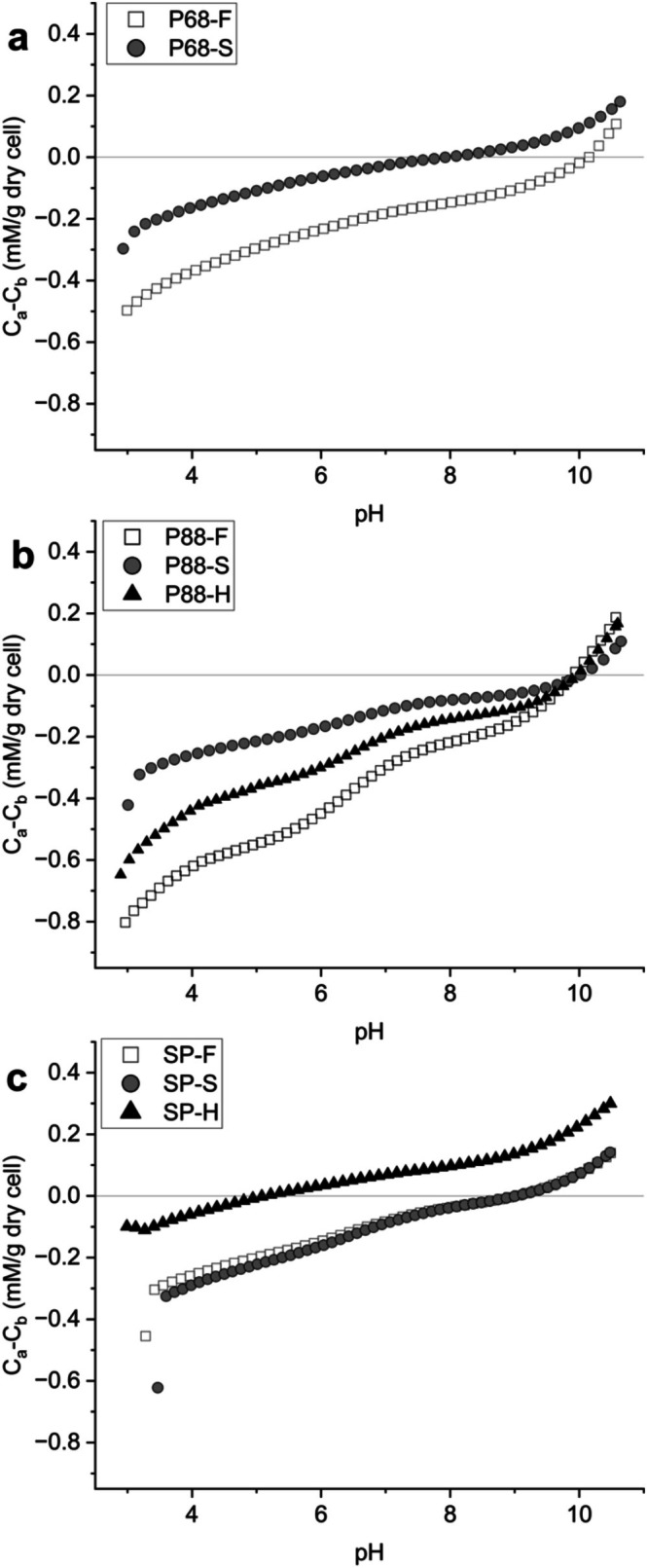
Net surface charge and point of zero charge (PZC) of cyanobacterial cells under salinity treatments. Data are presented in form of charge excess (mM/g dry cell) for each of (a) *Syn*. PCC6803 (P68), (b) *Syn*. PCC8806 (P88) and (c) *Spirulina platensis* SP. Symbols correspond to the average of titration data under each of freshwater (–F, white square), saline (–S, grey circle) and hypersaline (–H, black triangle) conditions. The PZC is the pH at which the net surface charge equals zero, indicating equal abundance of acidic and basic sites.

The compositional changes were reflected in the surface charge behaviour across pH. P68‐S exhibited a more positive surface charge than P68‐F across pH 3–10 (Figure 2a). The point of zero charge (PZC) decreased from pH 10.1 in P68‐F to 7.9 in P68‐S, consistent with the observed depletion of acidic groups. PCA of the LPM‐derived ligand concentrations further distinguished P68‐S and P68‐F based on shifts in functional group abundance and pKa distribution (Figure S3). Functional group pKa values formed distinct clusters around P68‐F or P68‐S in PCA space, demonstrating statistical differences in the protonation behaviour and surface group expression of each functional group based on treatment. Together, the ligand abundance and surface charge results indicate that salinity exposure in *Syn*. PCC6803 reduces the abundance of acidic surface functional groups, decreases carboxyl and hydroxyl pKa distributions and shifts the net surface charge towards more positive values. The chemical shifts show a restructuring of surface chemistry that could decrease the cell's capacity for ion binding and mineral interactions.

#### 3.1.2 Functional Groups and Lipid Chemistry

Across all species, ATR‐FTIR spectra exhibited distinct peaks corresponding to key surface functional groups that were modified by salinity acclimatisation (Figures 3 and S6–S14).

**FIGURE 3:**
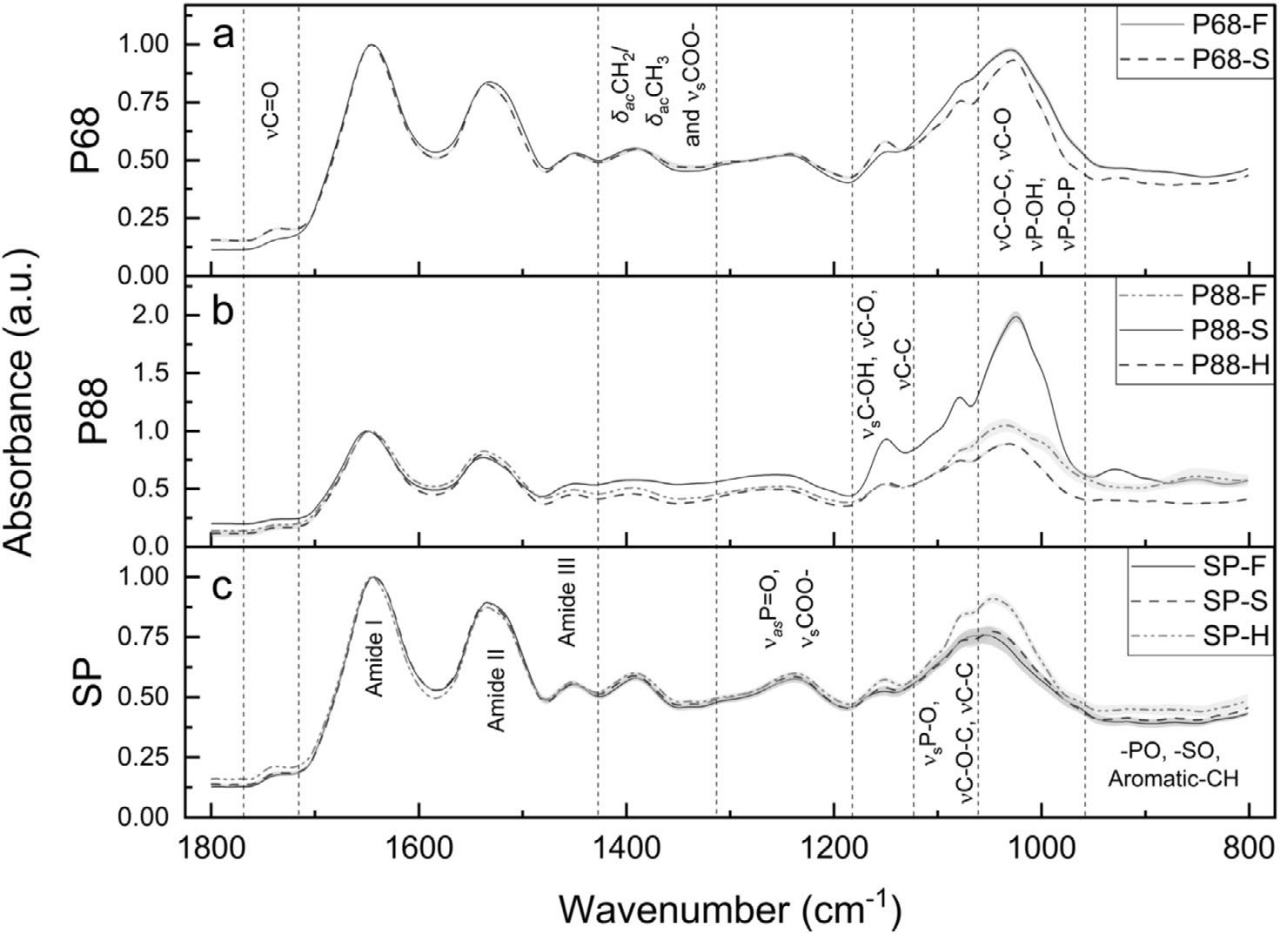
Averaged FTIR fingerprint region spectra of (a) *Syn*. PCC6803, (b) *Syn*. PCC8806 and (c) *Spirulina platensis* grown in freshwater (–F), saline (–S) and hypersaline (–H) culture conditions, collected as freeze‐dried cell powder. Key spectral regions highlighted include ~1740 cm^−1^ (νC═O of esters), ~1650 cm^−1^ (amide I), ~1540 cm^−1^ (amide II), ~1455–1300 cm^−1^ (amide III), 1388 cm^−1^ (*δ*_*ac*_CH2/*δ*_*ac*_CH3 and ν_*s*_COO^−^), ~1149–915 cm^−1^ (carbohydrates and polysaccharides), ~1078–915 cm^−1^ (phosphates and carbohydrates) and < 915 cm^−1^ (–PO, –SO and aromatic –CH). See Table 2 for specific band assignments and Figure S6–S14 for detailed fingerprint and lipid spectra.

The most prominent bands included: lipid *v*CH_2_ stretching vibrations near ~2925 and ~2855 cm^−1^ and lipid *v*CH_3_ stretching vibrations near ~2960 and ~2875 cm^−1^ typically associated with membrane lipid order and saturation; ester *v*C═O stretching near 1740 cm^−1^, indicative of lipids and membrane‐bound esters; amide I and II bands near 1650 and 1540 cm^−1^, reflecting protein backbone and surface‐associated peptides; and asymmetric stretching of *v*P═O in the ~1260–1230 cm^−1^ range and symmetric *v*P–O stretching at ~1078 cm^−1^ representing phosphoryl groups in the phosphodiester backbone of nucleic acids, proteins, lipids and possible polyphosphate. Polysaccharide‐rich regions were found between ~1150 and ~915 cm^−1^, with peaks near ~1150 and 1025 cm^−1^ assigned to *v*C–O and *v*C–O–C stretching of carbohydrates, overlapping with some phosphate peaks. Additional bands of interest included 1455 cm^−1^ of *δ*_*ac*_CH_2_/*δ*_*ac*_CH_3_ in proteins and lipids, *ν*_s_COO^−^ carboxylate stretching near 1388 cm^−1^ of carboxylic groups and aromatic or phosphoryl‐associated modes around 895–869 cm^−1^. A more comprehensive list of vibrational band assignments based on established signatures in literature can be found in Table 2. The spectral assignments provide a framework for interpreting species‐specific salinity responses.

**TABLE 2:** FTIR peak assignments.

Wavenumber, cm^−1^			Assignment (Figure 3 placement)	Comments	Citations
P68	P88	SP			
1797–1771			*ν*C═O of ester functional groups in lipids		1
1743–1734	1737–1734	1737	*ν*C═O of ester functional groups and possible protonated carboxylic groups	Position varies in literature	2, 3, 4, 5
1646	1648–1646	1646–1643	*ν*C═O of amides associated with proteins (~1650)	Position varies in literature. Also known as the Amide I Band	1, 6
1534–1531	1540–1537	1534	*δ*N–H of amides associated with proteins (~1540)	Position varies in literature. Also known as the Amide II Band	1, 6
1454–1449	1452–1449	1452	CH_3_ scissoring, *δ*_*ac*_CH_2_/*δ*_*ac*_CH_3_ in proteins and lipids (~1470, 1455, 1455–1300)	Position varies in literature. Common in peptidoglycan, teichoic acids, long alkyl chains, lipopolysaccharides and phospholipids. Also known as the Amide III band	2, 3, 5
1395–1389	1394	1391	*δ*_*ac*_CH2/*δ*_*ac*_CH3 and *ν*_*s*_COO^−^ of carboxylic groups (~1400, 1388)	Position varies in literature. Corresponds to deprotonated carboxyl surface sites	2, 3
1243–1241	1263–1246	1238	*ν*_*as*_P═O of phosphoryl groups present in the phosphodiester backbone of nucleic acids (DNA and RNA); possible phosphorylated proteins and polyphosphate (~1242); *ν*_*s*_COO– of ester groups (~1238); stretching of C–O of –COOH groups (~1260)		1, 2, 3, 5,
1149	1152–1149	1152	*ν*_*s*_C–OH, *ν*C‐O, *ν*C‐C from polysaccharides and carbohydrates (~1200–900)	Increase may be due to detection of esters in lipids (Da Rós et al. 2013)	5, 8, 9
1078 Shoulder	1078	1075	*ν*_*s*_P–O of phosphoryl groups present in the phosphodiester backbone of nucleic acids (DNA and RNA), possible phosphorylated proteins and polyphosphate; *ν*C–O–C and *ν*C–C bonds in polysaccharides (~1150–950; 1080; 1079–1038)	Position varies in literature. Polysaccharides include glycogen and peptidoglycan, uronic acids	2, 5, 10
1047–924	1035–930	1047–915	*ν*C–O–C, *ν*C–O of polysaccharides, *ν*P–OH and *ν*P–O–P of phosphate groups in phosphate oligomers	Polysaccharides include glycogen and peptidoglycan	2, 5, 8
898–858	852–849	895–869	–PO, –SO and aromatic –CH stretching vibrations		11, 12

*Note:* References for peak assignments: (1) Stehfest et al. (2005), (2) Dittrich and Sibler (2006), (3) Yee et al. (2004), (4) Da Rós et al. (2013), (5) Yuxia et al. (2016), (6) Yamanari et al. (2004), (7) Çelekli and Bozkurt (2011), (8) Kansiz et al. (1999), (9) Ferreira et al. (2011), (10) Shen et al. (2021), (11) Kaplan Can et al. (2019), (12) Dotto et al. (2012).

Salt‐treated P68‐S exhibited peak amplitudes that were more pronounced and narrower for esters (~1740 cm^−1^), polysaccharides (~1149 cm^−1^) and overlapping phosphate‐carbohydrate regions (~1078 and ~1030 cm^−1^, Figure 3aC–D) compared to the freshwater‐grown P68‐F (Figure 3a). In contrast, the 895–869 cm^−1^ band was more pronounced in P68‐F.

The *R*_3/2_ values, representing the CH_3_/CH_2_ intensity ratio, were 0.64 for P68‐F and 0.51 for P68‐S (Table S8). The lower *R*_3/2_ value in P68‐S shows a higher relative abundance of CH_2_ (methylene chains) compared to CH_3_ (terminal methyl groups). The spectroscopic results indicate that salt‐acclimatised *Syn*. PCC6803 cells exhibit an increase in lipid saturation and carbohydrate content and a concurrent depletion of phosphoryl‐rich groups.

#### 3.1.3 Surface Macromolecule Shifts

**FIGURE 4:**
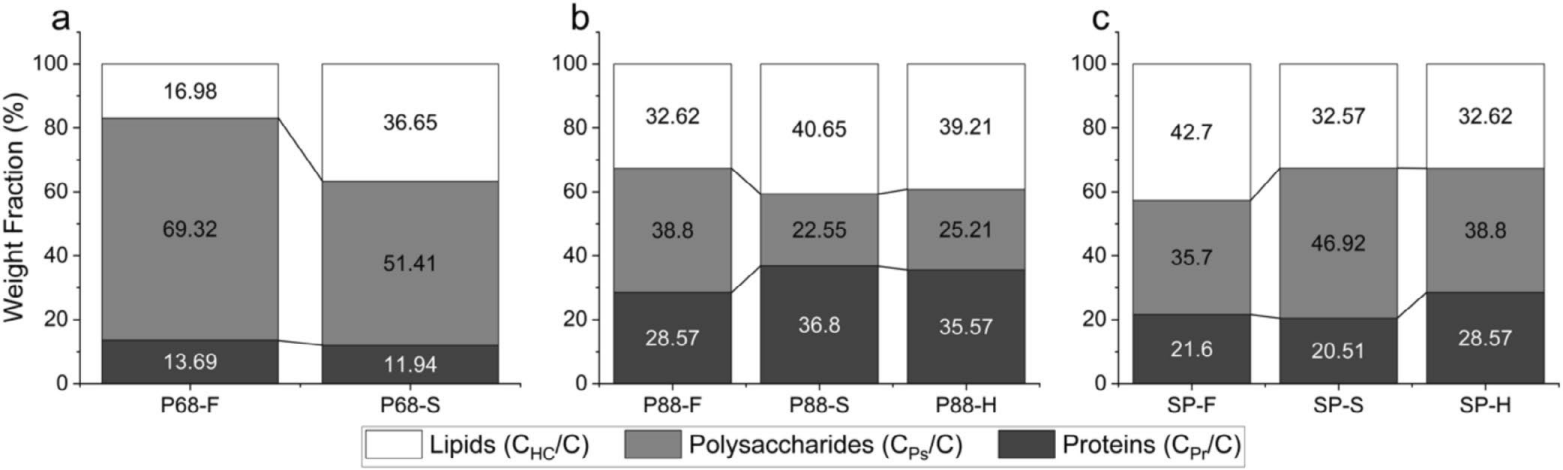
XPS data for hydrocarbon‐like (C_HC_/C), polysaccharide (C_Ps_/C) and protein (C_Pr_/C) weight fractions detected on cell surfaces for (a) P68, (b) P88 and (c) SP after growth in freshwater (–F), saline (–S) and hypersaline (–H) conditions.

XPS elemental analysis confirmed a pronounced surface chemistry shift in P68‐S. Compared to P68‐F, the overall protein‐derived carbon fraction (C_Pr_/C) decreased slightly (14%–12%), while the polysaccharide carbon fraction (C_Ps_/C) increased by 18% (69%–51%) (Figure 4a). In contrast, the hydrocarbon‐like carbon fraction (C_HC_/C) almost doubled (17%–37%), indicating enrichment of aliphatic moieties at the cell surface. Elemental ratios reflected a similar trend for P68‐S, where O/C dropped from 0.62 to 0.47 and N/C from 0.04 to 0.03 (Table 3). The loss of oxygenated and nitrogen‐rich related bonds is consistent with the titration‐inferred depletion of phosphoryl and carboxyl moieties (Table 1) and the FTIR‐detected reduction in phosphate‐related peaks (Figure 3a).

**TABLE 3:** Energy (eV) of XPS spectral bands detected on cells, mass fractions (%) and band assignments based on Paulo et al. (2018).

	Peak (eV)			Ratio		Mass fraction (%)								
						Carbon				Oxygen			Nitrogen	
	Total C	Total N	Total O	O/C	N/C	C1s	C1s A	C1s B	C1s C	O1s	O1s A	O1s B	N1s	N1s A
P68	285.06 ± 0.41	399.14 ± 0.84	531.75 ± 0.64											
P68‐F				0.62	0.04	30.8	13.5	43.9	11.8	56.5	21.1	22.4	91.7	8.3
P68‐S				0.47	0.03	22.4	29.8	25.5	22.3	62.5	19.7	17.8	96.9	3.2
P88	284.73 ± 0.06	399.67 ± 0.07	531.81 ± 0.16											
P88‐F				0.42	0.08	33.2	28.2	26.9	11.8	33.8	18.6	47.6	96.3	3.8
P88‐S				0.31	0.10	24.9	43.9	12.6	18.7	51.7	19.0	29.3	95.6	4.4
P88‐H				0.33	0.10	37.2	43.8	5.7	13.3	46.6	15.6	37.8	95.4	4.6
SP	284.78 ± 0.02	399.72 ± 0.04	532.16 ± 0.20											
SP‐F				0.37	0.06	26.8	36.3	18.8	19.1	33.6	22.1	44.3	95.7	4.3
SP‐S				0.46	0.06	34.3	30.8	22.2	12.7	26.8	24.7	48.5	96.4	3.7
SP‐H				0.42	0.08	33.2	28.2	26.9	11.8	33.8	18.6	47.6	96.3	3.8
Assignments						C–(C,H)	C–(O,N)	C═O; O–C–O	O═C–OH/C═O	O═C; P═O, P–O–Ring	C–OH; C–O–C; P–OH	HO–C	Unprotonated amine/amide	Protonated amine/amide
						Amino acids or lipids	Ether, alcohol, amine and amide	Carboxyl, amide, acetyl, hemiacetal	Carboxyl, esters	Esters, amides, phosphates	Hydroxyl, acetyl and hemiacetal	Hydroxyl		

At the bond level, P68‐S showed decreased contributions from C–(C, H) and O–C–O and an increase in C═O and O═C–OH bonds, suggesting a restructuring towards more carbonyl‐rich compounds, possibly from lipid oxidation or hydrolysis of phospholipids. Oxygen‐associated bonds also showed reduced C–OH, C–O–C and P–OH signals. Nitrogen bonding patterns revealed a relative increase in unprotonated amine/amide groups and a decrease in protonated forms, consistent with restructuring of surface peptide composition or protein structure.

Together, ligand abundance, surface charge model and spectroscopic data indicate that *Syn*. PCC6803 responds to salinity stress by decreasing phosphate‐ and protein‐rich bonds and increasing hydrocarbon‐ and polysaccharide‐like components. The XPS findings align with FTIR (Figure 3a) and PT results (Table 1), supporting the observation of a reorganisation of surface functional groups and macromolecular structure in this freshwater strain.

### 3.2 Surface Chemical Responses of *Syn.* PCC8806 (P88)

#### 3.2.1 Surface Site Modelling

**FIGURE 5:**
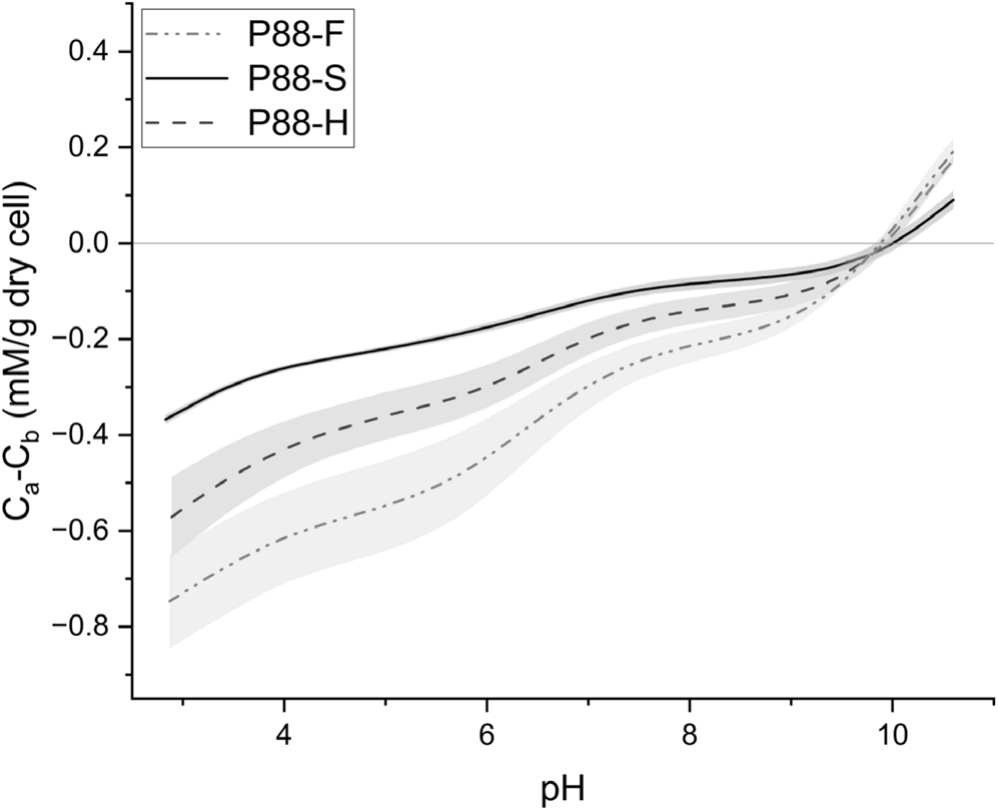
Representative image of potentiometric titration models created using LPM for P88 treatments under freshwater (–F), saline (–S) and hypersaline (–H) conditions. Models for P68 and SP treatments are displayed in Figure S2.

PT and LPM revealed differences in shifts in surface functional group composition for *Syn*. P8806 when grown in either freshwater or hypersaline conditions compared to standard saline. Both P88‐F and P88‐H showed an increase in concentrations of carboxyl, phosphoryl, amine and hydroxyl groups (Table 1). In P88‐F, phosphoryl groups more than tripled, while in P88‐H, carboxyl groups tripled and phosphoryl groups doubled. The increases in acidic functional group abundance corresponded with a more negative surface charge across the pH range of 3 to 10 for both P88‐F and P88‐H, with P88‐F exhibiting the most negative charge (Figure 2b), up to 0.37 C_a_‐C_b_ (mM/g_dry cell_) more negative than P88‐S. At pH > 10, both treatments shifted towards a more positive surface charge relative to P88‐S (Figure 5).

Modelling of pKa distributions revealed carboxyl groups in P88‐H clustered around pH 3.6, while P88‐F and P88‐S showed broader distributions (Figure 1b). Modelling of pKa distributions highlighted treatment‐specific changes in functional group acidity. In P88‐H, carboxyl sites clustered around pKa ≈ 3.6, whereas in P88‐F and P88‐S, carboxyl groups were more broadly distributed (Figure 1b). PCA of LPM‐modelled ligand concentrations further distinguished treatments, with phosphoryl groups in P88‐F enriched at mid‐range pKa values (~6.0–7.5) (Figure S4d). Hydroxyl groups in P88‐F were broadly distributed, whereas in P88‐H and P88‐S they clustered near pKa ≈ 10.3–10.5, demonstrating compositional or structural differences in high‐pKa surface groups.

Despite the shifts in functional group abundance and distribution, PZC remained consistent across all treatments (~9.9–10.0), implying a balanced adjustment in acidic and basic sites. Overall, *Syn*. PCC8806 exhibited a salinity‐dependent increase in functional group abundance, particularly for phosphoryl and carboxyl moieties, which increased relative negative charge with decreasing pH by up to 0.37 C_a_‐C_b_ (mM/g_dry cell_) for the F‐lineage.

#### 3.2.2 Functional Groups and Lipid Chemistry

ATR‐FTIR spectra revealed notable shifts in *Syn*. PCC8806 surface chemistry across salinity treatments (Figures 3b and S10). P88‐S exhibited the highest absorbance intensities across major vibrational regions, including ester *v*C═O stretching (~1740 cm^−1^), lipid *δ*_*ac*_CH_2_/*δ*_*ac*_CH_3_ bending (1455 cm^−1^), carboxylic ν_*s*_COO^−^ stretching (~1395 cm^−1^), phosphate‐related νP═O and νP–O stretching (~1260–1230, ~1078 ~1024 cm^−1^) and polysaccharide νC–O/νC–O–C stretching vibrations (~1149, ~1078, ~1024 cm^−1^). Aromatic, phosphate and sulphur‐containing moieties (–PO, –SO, aromatic C–H) followed a similar trend where the peak at 930 cm^−1^ was strongest in P88‐S, whereas the 850 cm^−1^ shoulder was comparable in P88‐S and P88‐F but weaker in P88‐H. By contrast, P88‐F and P88‐H showed reduced peak intensities across these regions, indicating loss or reorganisation of surface biopolymers. Between the salinity treatments, P88‐F generally retained greater absorbance than P88‐H for key carbohydrate, phosphate and carboxyl bands.

The *R*_3/2_ ratio also varied noticeably between treatments: 0.31 for P88‐S compared to 0.60 and 0.57 for P88‐F and P88‐H, respectively (Table S8). The low *R*_3/2_ value in P88‐S indicates a greater abundance of methylene groups. In contrast, F‐ and H‐lineages demonstrated higher CH_3_/CH_2_ ratios.

#### 3.2.3 Macromolecule Shifts

XPS analysis revealed that culturing under freshwater conditions impacted the surface structure of *Syn*. PCC8806 more than hypersaline conditions relative to the standard saline control across the C_HC_/C, C_Ps_/C and C_Pr_/C fractions (Figure 4b). For the P88‐F treatment, the C_HC_/C fraction decreased by 7.9% (40.7%–32.6%), the C_Ps_/C increased by 16.3% (22.5%–38.8%) and the C_Pr_/C fraction decreased by 8.2% (36.8%–28.6%). In contrast, P88‐H showed only minor shifts (~1%–3%) across these macromolecular fractions. The changes were mirrored in the elemental ratios: O/C increased from 0.31 in P88‐S to 0.42 in P88‐F, while N/C decreased from 0.10 to 0.08, consistent with enriched oxygen‐rich polysaccharide content and reduced protein abundance under freshwater conditions. Elemental ratios in P88‐H remained closer to P88‐S, where O/C shifted from 0.31 to 0.33 and the N/C ratio remained the same, showing limited deviation from the native saline state.

PT confirmed an increase in functional group abundance in P88‐F and P88‐H. Both treatments showed increased C–(C,H) and C═O bonds and decreased O═C–OH bonds (Table 3). In P88‐F, decreases in C–(O,N) and O–C–O bonds align with relatively lower protein content and elevated polysaccharide weight fraction signals, while in P88‐H, there is an increase in O–C–O bonds but a lack of change in the overall protein weight content. Oxygen spectra revealed losses in amide‐ and ester‐associated bonds in both treatments. Minimal changes in nitrogen bond fractions (< 1%) show that the observed decrease in protein‐like signal in P88‐F is aligned with polysaccharide expression rather than significant protein degradation, as demonstrated by consistent amide band stability in the FTIR spectra.

Overall, the XPS analysis indicates that while both treatments alter surface biochemistry, freshwater exposure drives greater polysaccharide enrichment and lipid remodelling, whereas hypersaline conditions primarily induce restructuring of surface macromolecules.

In summary, Syn. PCC8806 surface remodelling under freshwater conditions is characterised by increased polysaccharide expression, altered lipid composition and reduced protein signatures. Hypersaline exposure induces more conservative changes, preserving much of the native macromolecular architecture observed under standard salinity. The XPS findings complement the surface site modelling (Figures 1b and 2b) and FTIR results (Figure 3b), reinforcing that *Syn*. PCC8806 displays salinity‐dependent flexibility in its cell envelope chemistry, with greater changes in freshwater than hypersaline environments.

### 3.3 Surface Chemical Responses of *S. platensis* (SP)

#### 3.3.1 Surface Site Modelling

PT and LPM showed clear salinity‐dependent shifts in the surface chemistry of *S. platensis* . SP‐F and SP‐S cultures displayed similar charge curves, although SP‐S was slightly more negative below pH 6 (Figure 2c). In contrast, SP‐H cultures carried a more positive charge from pH 3–10, up to 0.38 C_a_‐C_b_ (mM/gdry cell) more positive than the F‐lineage and 0.24 C_a_‐C_b_ (mM/gdry cell) more positive than the S‐lineage. Changes in charge were mirrored by functional group concentrations (Table 1). Relative to SP‐F, SP‐S contained higher levels of carboxyl (+69.3%), but fewer amines (−35.4%), explaining its negative charge at low pH. SP‐H showed the opposite trend: negatively charged phosphoryl sites fell by 55.6%, with minor changes to carboxyl, amine and hydroxyl groups, yielding a net positive surface at most pH values.

Functional group distributions also shifted between treatments. PCA of the LPM‐derived site densities (Figure S5) linked the broad carboxyl pKa spread in SP‐S (pH ≈ 3.8–5.8) to its elevated carboxyl content, whereas phosphoryl‐associated pKa values clustered with SP‐F and SP‐S, consistent with the loss of phosphate in SP‐H. Amine‐rich pKa values grouped with SP‐F and SP‐H, reflecting the amine decrease in SP‐S. Hydroxyl site distributions were more clustered in SP‐H at pH ≈ 10, while SP‐F and SP‐S showed similar hydroxyl spreads from pH 9–10.5. Interestingly, the PZC fell from pH 9.0 in SP‐F and 8.9 in SP‐S to 4.8 in SP‐H, underscoring the reduction in surface phosphate under saline stress. Overall, *S. platensis* responds to marine salinity by accumulating acidic sites, especially carboxylates, whereas hypersalinity strips phosphoryl groups and shifts the surface towards a more positive charge, up to 0.38 C_a_‐C_b_ (mM/gdry cell) more positive than the F‐lineage.

#### 3.3.2 Functional Groups and Lipid Chemistry

ATR‐FTIR analysis revealed distinct shifts in surface chemical composition in *S. platensis* across salinity treatments (Figure 3c). Hypersaline‐treated cells exhibited the highest peak amplitudes for ester *v*C═O stretching (~1737 cm^−1^), polysaccharide *ν*C–O/*ν*C–O–C stretching vibrations (~1152, ~1075, ~1047 cm^−1^) and surface moieties including overlapping –PO, –SO and aromatic –CH functional groups (~910, ~870 cm^−1^). The ester, polysaccharide, phosphate, sulphur and aromatic signals were consistently higher than those observed under both freshwater and saline conditions.

The *R*_3/2_ ratio decreased progressively with salinity: from 2.10 in SP‐F, to 1.87 in SP‐S and 1.13 in SP‐H (Table S8). Notably, *S. platensis* exhibited higher *R*_3/2_ values overall compared to the other two species. Together, the spectroscopic data show that *S. platensis* responds to hypersaline conditions by increasing the abundance of ester‐linked and carbohydrate‐rich moieties at its surface.

#### 3.3.3 Macromolecule Shifts

XPS analysis revealed salinity‐induced modifications in the macromolecular surface composition of *S. platensis* (Figure 4c). In SP‐S, the C_Ps_/C fraction increased from 36% in SP‐F to 47%, while the C_HC_/C fraction decreased from 43% to 33%. The C_Pr_/C fraction remained relatively stable at 22% in SP‐F versus 21% in SP‐S. For SP‐, the C_Pr_/C fraction increased to 29%, C_Ps_/C remained similar to SP‐S (39%) and C_HC_/C also decreased to 33%. Elemental ratios further supported weight fraction changes (Table 3). The O/C ratio increased from 0.38 in SP‐F to 0.46 in SP‐S and 0.42 in SP‐H, indicating an overall enhanced polysaccharide expression. The N/C ratio remained stable (0.06) between SP‐F and SP‐S but rose to 0.08 in SP‐H.

Analysis of carbon bond types showed an increase in C–(C,H), C═O and C–O–C bonds in both saline and hypersaline treatments, along with a decrease in C–(O,N) and carboxylic O═C–OH bonds (Table 3). The changes in bond fractions suggest enhanced lipid branching and reduced surface protein carboxylation. In the oxygen region, SP‐S showed decreased O═C, P═O and P–O–ring signals but increased C–OH, P–OH and HO–C fractions. SP‐H followed a similar trend, though with more stable or increased phosphoryl‐related signals. Nitrogen analysis showed a shared pattern of increased unprotonated amine/amide fractions and decreased protonated ones. Together, these data show that *S. platensis* modifies both polysaccharide and protein surface content in response to salinity, with greater interpreted protein incorporation and surface lipid restructuring under hypersaline stress.

Together, the ligand abundance, surface charge modelling and spectroscopic data show that *S. platensis* modifies both surface polysaccharides and proteins in response to increasing salinity. Marine conditions prompt a carbohydrate‐enriched surface, while hypersaline exposure leads to elevated protein content and increased lipid saturation. Table 4 synthesises inter‐specific differences in salinity acclimatisation responses.

**TABLE 4:** Side‐by‐side comparison of long‐term salinity acclimatisation responses in the three study strains.

	*Synechocystis* sp. PCC6803 (P68)	*Synechococcus* sp. PCC8806 (P88)	*Spirulina platensis* (SP)
Native setting and baseline medium (salinity, ‰)	Freshwater: BG‐11 (~2)	Marine: ASN‐III (~30)	Alkaline soda lake: Zarrouk's (~10)
Salinity treatments tested (‰ NaCl)	2, 30	6, 30, 60	10, 30, 60
Key functional‐group shifts^a^	30‰: ↓ phosphoryl, carboxyl, hydroxyl ↑ amine	6‰: ↑ phosphoryl (3×) 60‰: ↑ carboxyl (×3)	30‰: ↑ carboxyl, ↓ amine 60‰: ↓ phosphoryl (−56%), ↑ ester and protein
Net surface‐charge trend (pH 3–10)	30‰: more positive	6‰: more negative 60‰: more negative	30‰: slightly more negative 60‰: markedly more positive
PZC shift (ΔpH)	30‰: −2.2 (10.1 → 7.9)	6‰: ~0 (stable 9.9–10.0) 60‰: ~0 (stable 9.9–10.0)	30‰: ~0, stable 60‰: −4.2 (9.0 → 4.8)
Lipid *R*_3/2_ ^b^ signal	30‰: *R*_3/2_ 0.64 → 0.51 ⇒ ↓ fluidity, ↑ saturation	6‰: *R*_3/2_ 0.31 → 0.60 ⇒ ↑ fluidity, ↓ saturation 60‰: *R*_3/2_ 0.31 → 0.57 ⇒ ↑ fluidity, ↓ saturation	30‰: *R*_3/2_ 2.10 → 1.87 ⇒ ↓ fluidity, ↑ saturation 60‰: *R*_3/2_ 2.10 → 1.13 ⇒ ↓ fluidity, ↑ saturation
Inferred membrane‐remodelling strategy^c^	Replace P‐lipids with glycol/neutral lipids; conserve P and decrease membrane fluidity to curb Na^+^ influx	Enrich PG and adjust charge while preserving bulk lipids; maintain photosynthetic capacity across salinity span	Two‐stage ‘threshold’ model: moderate salt adds osmoprotectants; hypersalt triggers P‐lipid replacement & further saturation to stabilise membrane

^a^ Relative to the freshwater (P68), marine (P88) or alkaline (SP) baseline treatment.

^b^ *R*_3/2_ = CH_3_/CH_2_ FTIR area ratio; lower values → longer/more saturated chains, lower fluidity.

^c^ Summarises integrated FTIR, XPS and titration evidence (see Discussion 4.1–4.3).

## 4 Discussion

### 4.1 Salinity‐Induced Surface Remodelling Reveals Functional Group Shifts and Membrane Modifications Across Bacteria

Surface‐sensitive analyses (FTIR, PT and XPS) revealed species‐specific responses to salinity stress. In freshwater *Syn*. PCC6803, salt acclimatisation resulted in a lower PZC, more positive surface charge across pH and decreased abundance of negatively charged surface groups such as phosphoryls. FTIR data showed a relative increase in polysaccharide‐associated signals in saline conditions (Figure 3a and Table 2), consistent with the upregulation of membrane‐bound GG, a known osmoprotectant produced by *Syn*. PCC6803 under salt stress (Huang et al. 2006). Transport and signal proteins are also membrane‐bound on the cell surface and drive downstream metabolic activity as a response to environmental change (Babele et al. 2019). XPS data supported these trends by showing increased O/C and N/C ratios, suggesting adjustments related to protein expression, photosynthetic responses and metabolic activity as a result (Table 3) (Zhao et al. 2015). Kokilathasan et al. (2025) reported similar changes to elemental composition and O/C and N/C ratios in response to nanoplastic exposure, suggesting that surface chemical reorganisation may be a common response to environmental perturbation. Spectroscopic analysis also revealed an increase in ester signals and a decrease in the *R*_3/2_ ratio (Table S8) under saline conditions, consistent with lipid remodelling involving more linear and saturated fatty acid chains (Igisu et al. 2009) and a potential reduction in membrane lipid diversity (Harayama and Riezman 2018). The increase in lipid saturation contributes to reduced membrane fluidity, which has further implications in surface signalling and permeability dynamics (Los and Mironov 2015). Thus, the interpreted shift in surface lipid composition could potentially be linked to the production or externalisation of glycolipids or neutral lipids during acclimation.

The interpretation of decreased membrane fluidity contrasts with earlier findings of increased synthesis of acyl‐lipid fatty acid desaturase enzymes (Los and Mironov 2015) and increased membrane fluidity under short‐term saline exposure in *Syn*. PCC6803 (Allakhverdiev et al. 1999). Increased membrane fluidity promotes impacts the activation and biosynthesis of the Na^+^/H^+^ antiport system to maintain ionic balance and prevent photosynthetic suppression from ionic stress (Allakhverdiev et al. 2001). The discrepancy may stem from methodological differences: Allakhverdiev et al. (1999) assessed salt shock for 30 h in freshwater‐cultured cells, while our study examined long‐term acclimatisation to salinity‐adjusted growth conditions. Our findings suggest that prolonged exposure may instead favour structural membrane stabilisation via saturation, rather than fluidity enhancement for ion extrusion.

A broader understanding of the acclimatisation process in *Syn*. PCC6803 comes from Marin et al. (2004), who identified a two‐phase response to salt stress. The initial phase involves a rapid upregulation of numerous genes and the production of salt‐response proteins within the first hour. The initial response is followed by a long‐term maintenance of a subset of salt‐response genes after 24 h. It is possible that the sustained synthesis of GG and transport proteins is sufficient to maintain ion homeostasis (Mansour 2013) despite the decline in membrane fluidity and Na^+^/H^+^ antiport activity (Allakhverdiev et al. 1999). We thus propose that the long‐term salt acclimatisation strategy for *Syn*. PCC6803 may be to buffer stress by strategically remodelling the membrane to reduce ion permeability even at the cost of metabolic efficiency.

Similar patterns of membrane adaptation were observed in *S. platensis* , which progressively restructured its membrane in response to increasing salinity. At moderate salinity, the strain exhibited elevated carboxyl group abundance and a decrease in amine groups, while maintaining a relatively stable net surface charge above pH 6. Spectroscopic data also indicated a trend towards increased lipid saturation and suggested a rise in surface‐associated polysaccharide and protein content. Under hypersaline conditions, further remodelling was evident: phosphoryl group abundance declined, surface charge shifted more positive and ester‐, polysaccharide‐ and protein‐related signals became more pronounced. As in *Syn. PCC6803*, the enhanced polysaccharide signals are consistent with the accumulation of plasma membrane (PM)‐bound GG as an osmoprotectant (Markou et al. 2023). The interpretation of osmo‐protectant synthesis is supported by prior studies showing increased carbohydrate content and the accumulation of GG and trehalose in salt‐exposed *S. platensis* (Volkmann et al. 2008; Çelekli et al. 2016). The increased amino‐ and polysaccharide‐associated signals also align with the upregulation of proteins involved in fatty acid desaturation, osmotic regulation, ABC transport and signal recognition (Wang et al. 2013), as well as transporter and photosynthetic proteins that support ion homeostasis under salinity stress (Ismaiel et al. 2018).

The 11% drop in the *R*_3/2_ ratio from the freshwater to marine salinity treatment in *S. platensis* indicates a shift towards a more saturated lipid profile as salinity rose. However, this pattern contradicts reports of lower saturation, higher polyunsaturated fatty acid (PUFA) content and increased membrane fluidity in *Spirulina* grown at elevated salinity (Bhakar et al. 2013; Markou et al. 2023). The most likely reason is that the cultures in our study were maintained at 30°C, which is below the optimal 35°C–38°C range for this species (Vonshak 1997) and temperature strongly modulates fatty‐acid desaturation. Indeed, *S. platensis* shows marked intraspecific variability in PM composition that depends on growth medium and culture regime (Çelekli et al. 2016; Aouir et al. 2017). Controlled studies demonstrate that higher temperature and stronger light generally drive cells towards greater saturation, whereas cooler, lower‐light conditions favour PUFA accumulation (Aouir et al. 2017). Consistent with culture‐specific growth dynamics, Mühling et al. (2005) found higher PUFA ratios at 20°C, matching the comparatively unsaturated lipid profile observed in SP‐F.

We propose that *S. platensis* used a threshold‐based membrane acclimatisation strategy under salinity stress. At moderate salinity, surface remodelling involves increased carboxyl and polysaccharide expression, some enrichment of surface‐bound osmoprotectants and early signs of lipid saturation and protein restructuring, while phosphoryl groups remain largely preserved. Under hypersaline conditions, more pronounced changes emerge, including a substantial decline in surface phosphoryl groups, enhanced expression of proteins and polysaccharides and further shifts in lipid composition. The remodelling pattern implies that *S. platensis* initially responds to salinity with protective surface modifications but crosses a physiological threshold at higher stress levels that require deeper restructuring of the membrane, including phosphoryl replacement. Similar to *Syn*. PCC6803, the long‐term acclimatisation response favours lower metabolic performance and reduced ion permeability over increased ion expulsion and photosynthetic activity. The threshold‐stage model aligns with previous findings of strain‐specific and condition‐dependent lipid remodelling in *Spirulina* and highlights the role of ecological context and stress intensity in shaping cyanobacterial surface responses.

In contrast to the previous species, the marine strain *Syn*. PCC8806 demonstrated consistent lipid and protein weight fractions at elevated salinity levels, suggesting minimal changes to XPS‐detected bulk membrane composition. Nonetheless, clear surface‐level restructuring was observed under both freshwater and hypersaline treatments, including modifications to the glycoprotein‐rich S‐layer. Under freshwater conditions, *Syn*. PCC8806 exhibited a more negative surface charge, a tripling of phosphate‐associated signal intensity, polysaccharide enrichment and decreased saturation of the lipid profiles, suggesting a more fluid membrane. The observations of surface remodelling are interpreted as consistent with enrichment of osmoprotectants and an enrichment of phosphate‐containing surface groups, particularly phosphatidylglycerol (PG), a major thylakoid membrane lipid (Lu et al. 2012). Given that high concentrations of Na^+^ disrupt electron transport in Photosystems I and II and reduce the efficiency of photosynthesis (Allakhverdiev et al. 2001), lower salinity conditions may promote the biosynthesis of PM lipid structures that enhance photosynthetic activity. For cyanobacterial species, photosynthesis is more efficient under low salinity conditions (Wada and Mizusawa 2009) and PG is a key component of the thylakoid membrane that supports efficient photosynthesis under low‐salinity conditions (Wada and Mizusawa 2009). Furthermore, decreased lipid saturation and improved membrane fluidity are necessary for the optimal function of thylakoid membranes and photosynthetic efficiency (Yamamoto 2016). Thus, we interpret PG enrichment in *Syn*. PCC8806 as a mechanism associated with low‐salinity conditions favourable for photosynthesis.

Under hypersaline conditions, *Syn*. PCC8806 exhibited increased carboxyl and phosphoryl group signals and a more negative surface charge, suggesting membrane restructuring and potential upregulation of salt‐stress proteins such as Na^+^/H^+^ antiporters (Tsunekawa et al. 2009). The similar lipid reorganisation and phosphoryl increase also suggest an increase in PG and photosynthetic activity as salinity increases despite increased ion permeability. Notably, the PZC remained consistent across all salinity treatments, suggesting a stable balance between total positively and negatively charged surface groups. Similar observations have been reported in the halophilic alga *Dunaliella salina* , which maintained a constant membrane lipid ratio when exposed to high salinity conditions (Peeler et al. 1989). It was proposed that the stability of the PM composition under a wide range of saline conditions is an adaptive mechanism for tolerating salinity stress (Peeler et al. 1989). Proteomic analyses of salt‐stressed *D. salina* further support PM stability by demonstrating the activation and regulation of membrane stabilisation, signal transduction and ion transport proteins (Katz et al. 2007), indicating that the internal salt‐response mechanisms likely compensate for the lack of change in lipid weight fraction. The parallels with *D. salina* suggest that in *Syn*. PCC8806, internal regulatory mechanisms and osmoprotectant synthesis, rather than shifts in bulk lipid composition, may play dominant roles in maintaining homeostasis under hypersaline conditions.

Therefore, *Syn*. PCC8806 appears to rely on surface‐level functional‐group adjustments and protein machinery, rather than extensive lipid turnover, to sustain homeostasis under high salt. We therefore propose a long‐term salt acclimatisation strategy based in salinity resilience, where it preserves core membrane architecture while fine‐tuning surface charge, PG abundance and stress‐response proteins to support both osmotic balance and continued photosynthetic capacity across a broad salinity spectrum.

Together, the surface remodelling findings reveal that long‐term salinity acclimatisation in non‐marine cyanobacteria involves a strategic shift towards membrane stabilisation through lipid saturation and phosphoryl group reduction, likely to conserve energy and limit Na^+^ influx. In contrast, the marine strain Syn. PCC8806 preserved membrane architecture while fine‐tuning surface charge and PG abundance to support photosynthesis across salinity gradients. These divergent strategies suggest that while all strains remodel their cell envelopes under salt stress, the balance between permeability control, metabolic efficiency and photosynthetic performance is finely tuned to each species' ecological niche.

### 4.2 Divergent Membrane Remodelling Strategies: Non‐Marine Strains Reduce Fluidity and Conserve Phosphorus While Marine Strains Enhance Photosynthetic Capacity Under Salinity Stress

Across all three cyanobacterial strains, salinity stress triggered detectable surface remodelling. However, the degree and nature of these changes suggest a combination of shared adaptive motifs and distinct, species‐specific strategies. All strains exhibited surface remodelling indicative of membrane adjustments, but the patterns may have diverged based on their native salinity niches.

Freshwater *Syn*. PCC6803 and alkaliphilic *S. platensis* reduced phosphoryl signals and showed stronger ester peaks, more positive surface charge and lower *R*_3/2_ ratios, implying phospholipid replacement, increased lipid saturation and decreased membrane fluidity. Although increased membrane fluidity supports ion export and photosynthesis efficiency (Allakhverdiev et al. 2001), increased lipid saturation is understood to enhance salt tolerance by reducing membrane permeability to NaCl (Molitor et al. 1990). Similar mechanisms have been documented in other species: the snow alga *Clamydomonas nivalis* increased saturation by 18.76% after 7 h of exposure to 0.75% NaCl (Lu et al. 2012), while the freshwater cyanobacterium *Anacystis nidulans* exhibited a 178% increase in fatty acid saturation when grown in 0.5 M NaCl (Molitor et al. 1990). Similarly, *D. salina* increased the sterol fraction of the PM lipids to 55% under exposure to 3.4 M NaCl (Peeler et al. 1989). The spectroscopic‐detected rise in esters in this study is consistent with the accumulation of esterified sterols and glycolipid‐linked fatty acids that serve to decrease membrane fluidity (López‐Lara et al. 2003; Mansour 2013).

In contrast, marine *Syn*. PCC8806 increased phosphoryl abundance and *R*_3/2_ ratios under both hypo‐ and hypersaline treatments while maintaining a consistently negative surface charge. These features point to PG enrichment that preserves membrane fluidity and supports photosynthetic electron transport when external Na^+^ levels fluctuate (Wada and Mizusawa 2009; Yamamoto 2016). The genus *Synechococcus* shows significant variation in fatty acid saturation and composition between species (Los and Mironov 2015). As a result, observations of salinity stress‐induced PM restructuring patterns of related *Synechococcus* species may not be the same as restructuring in marine strain *Syn*. *PCC8806* (Cañavate and Fernández‐Díaz 2022). Although the decreased saturation and increased membrane fluidity at higher salinities contradict the observed PM salinity response of another halophilic species, *D. salina* (Peeler et al. 1989), other organisms have also been observed to decrease lipid saturation as a salt‐tolerance mechanism. Documented species include microalga *Chlorella vulgaris* (Lu et al. 2009), cyanobacteria *Syn*. *PCC6803* (Allakhverdiev et al. 1999) and diatom *Nitzchia laevis* (Chen et al. 2008) among other photosynthetic microbes (Kumari et al. 2013) as well as plants such as broccoli root cells and others (López‐Pérez et al. 2009; Mansour et al. 2015). However, the stable PZC in P8806 echoes observations in *D. salina* where balanced charge is maintained by up‐regulating ion transport and membrane stabilisation proteins rather than altering lipid ratios (Peeler et al. 1989; Katz et al. 2007). Therefore, an overview of general adaptive patterns is preferable to understand microbial response to salinity stress.

**FIGURE 6:**
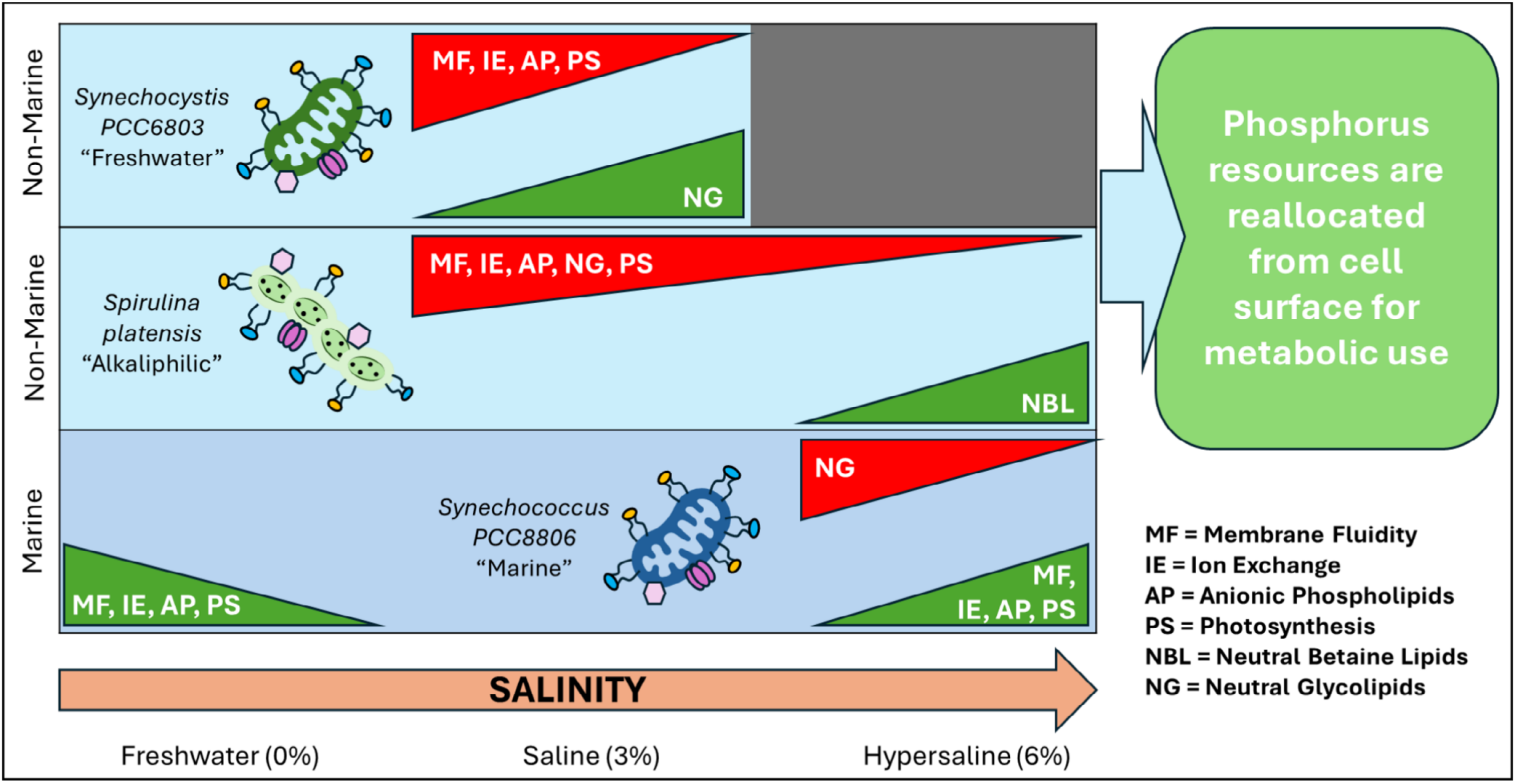
Visual summary comparing the dominant patterns of response to salinity for the three cyanobacterial strains. Triangle height and colour indicates relative change of parameters (read left‐to‐right with increasing salinity) based on functional group abundance, membrane lipid saturation and surface charge. Green represents relative increase and red represents relative decrease. Species‐specific strategies highlight membrane rigidity versus fluidity, phosphorus conservation and photosynthetic adaptations.

Our findings demonstrate that salinity‐induced restructuring of the cyanobacterial cell surface is closely tied to species‐specific differences in membrane composition and phosphorus utilisation strategies. Across all three strains, we observed shifts in functional group abundance, surface charge and lipid‐associated signals, suggesting that membrane remodelling plays a key role in acclimatisation to changing salinity (Figure 6). The results align with established models in which PM restructuring under salinity stress helps maintain cell integrity (Mansour et al. 2015), supports the synthesis of transport proteins and protective biomolecules (Mansour et al. 2003) and preserves signal transduction pathways (Mansour and Salama 2019). Overall, while all three cyanobacteria modulate phosphoryl content, lipid composition and surface charge under salinity stress, *Syn*. PCC6803 and *S. platensis* converge on strategies that reduce membrane fluidity and conserve phosphorus, whereas *Syn*. PCC8806 prioritises membrane fluidity and photosynthetic performance. Figure 6 provides a visual summary of the overall interpretations of salinity acclimatisation strategies between the studied cyanobacteria species. The differences in acclimatisation strategies reflect a continuum of adaptive strategies shaped by ecological niche, rather than a universal response.

### 4.3 Phospholipids: A Proposed Phosphorus Reservoir During Energy‐Intensive Salinity Acclimatisation

While prior studies have shown that phospholipid and glycolipid composition varies widely across cyanobacterial taxa, with no universal pattern linking salinity to lipid class distribution (Cañavate and Fernández‐Díaz 2022), our surface‐level analyses offer new insight into how biochemical strategies manifest externally. The observed changes in phosphoryl, ester and carbohydrate‐associated surface groups suggest that membrane remodelling is reflected in measurable surface chemistry. Changes in surface groups may arise from the reorganisation of specific lipid classes that differ in bilayer‐forming capacity and charge properties. For example, the balance between bilayer‐forming phospholipids such as phosphatidylcholine (PC) and PG, non‐bilayer‐forming lipids like phosphatidylethanolamine (PE) and signalling molecules such as phosphatidylinositol (PI) (Lu et al. 2012) likely influence membrane fluidity and functional group exposure. In addition, the substitution of phosphate‐containing lipids with non‐phosphorus glycolipids such as digalactosyldiacylglycerol (DGDG), sulfoquinovosyldiacylglycerol (SQDG) and monogalactosyldiacylglycerol (MGDG) or betaine lipids like diacylglyceryl‐*N*‐trimethylhomoserine (DGTS) (López‐Lara et al. 2003; Kumari et al. 2013), may explain the reduction in phosphoryl signals and the concurrent rise in ester and carbohydrate features. In particular, the replacement of anionic PG with SQDG under phosphorus‐limiting conditions (Oishi et al. 2022) offers a plausible biochemical basis for the decreased phosphoryl group signals observed in saline‐grown *Syn*. PCC6803 and hypersaline‐grown *S. platensis* . The lipid substitutions would maintain membrane charge balance and function while reallocating phosphorus for essential internal processes.

We thus propose a model in which salt‐stressed, non‐marine cyanobacteria remobilise membrane‐bound phosphorus as part of a broader metabolic adaptation. Under salt stress, cyanobacterial cells experience reduced photosynthetic efficiency due to Na^+^‐induced disruptions to Photosystems I and II (Allakhverdiev et al. 2001; Singh et al. 2022), alongside the upregulation of stress response genes (Fulda et al. 2006), salt‐stress proteins (Klähn et al. 2021) and energy‐demanding ion transporters such as the Na^+^/H^+^ antiport system (Gimmler 2000). Additionally, detoxification of ROS becomes essential under saline conditions (Yang et al. 2024), further taxing ATP reserves. In such metabolically demanding contexts, phosphorus becomes a limiting nutrient not only for ATP synthesis but also for nucleic acid production and stress protein biosynthesis (Vonshak 1997; Pandhal et al. 2008). We, therefore, propose that phosphate groups from membrane phospholipids are remobilised into the cytosol to meet internal energy demands, while the structural integrity of the membrane is maintained through the substitution of phosphate‐free lipids such as DGDG, SQDG or DGTS (López‐Lara et al. 2003; Gupta and Gupta 2021).

Such phospholipid replacement is a well‐documented phenomenon across diverse taxa under phosphorus limitation. In bacteria, the substitution of PG with DGTS or SQDG has been observed in the diazotrophic bacterium *Sinorhizobium meliloti* (López‐Lara et al. 2003), *Rhodobacter sphaeroides* (Benning et al. 1995) and alpha proteobacteria *Pseudomonas diminuta* (Minnikin et al. 1974). In eukaryotic phototrophs, oat plants under phosphate starvation replaced phospholipids with DGDG and sterol glucosides without loss of membrane functionality (Andersson et al. 2003, 2005), while the green alga *Chlorella kessleri* and purple photosynthetic bacterium *R. sphaeroides* demonstrated similar substitutions (Benning et al. 1995; Oishi et al. 2022). PG substitutions may also serve to reduce ion attraction and limit surface adsorption of toxic ions (Wagatsuma 2017). Notably, our study's spectroscopic findings of decreased phosphoryl signal, increased ester content and a shift towards more positive surface charge are consistent with documented lipid transitions and further suggest that this strategy may be widespread among non‐marine cyanobacteria. Even *Syn*. PCC6803 has also been observed to remobilise phosphorus from PG sources under phosphorus starvation (Hiyoshi et al. 2024) where phosphorus concentrations were completely removed compared to typical BG11 media concentrations of 0.2 mM P used in our study.

The phosphorus engine model may also explain why *Syn*. PCC8806, a marine strain with speculated more efficient osmotic regulation systems, maintains phosphoryl‐rich membranes and even increases PG content under salinity fluctuations. In contrast to *Syn*. PCC6803 and *S. platensis* , *Syn*. PCC8806 maintained a stable PZC and surface charge balance across salinity treatments, suggesting fewer constraints on phosphorus or energy availability. The increased PG content observed in *Syn*. PCC8806 under hypo‐ and hypersaline conditions may promote membrane fluidity and support enhanced photosynthetic activity under stress (Wada and Mizusawa 2009; Yamamoto 2016), avoiding the need for phosphorus reallocation. The membrane remodelling strategy suggests that species with inherently greater salinity tolerance may rely more on robust internal ion homeostasis and photosynthetic regulation, rather than sacrificing phosphorus‐containing lipids. The evidence of phosphorus reallocation underscores the importance of cellular energy economy and nutrient allocation in shaping species‐specific acclimatisation strategies.

However, several limitations to the proposed phosphorus engine model must be considered. First, the cellular pool of phosphorus‐containing lipids is finite and prolonged stress may still result in deficiency if external phosphate is unavailable. Second, the process of degrading phospholipids and synthesising alternative lipids, such as glycolipids or betaine lipids, still requires energy. Under stressful conditions, the energy demand for maintaining cellular functions and stress responses is already high, making biomolecule synthesis an additional burden (Vonshak et al. 1988; Pandhal et al. 2008). Third, while glycolipids and betaine lipids can replace phospholipids, it is not clear whether they can replace the functional properties of phospholipids. Insufficient replacement of functions can negatively affect membrane‐associated processes such as signalling, transport and enzyme activities if they cannot accomplish the same role long‐term. Finally, the phosphorus engine model represents an intermediate acclimatisation strategy that may support survival during stress exposure but not necessarily long‐term adaptation. As demonstrated by Dhiab et al. (2007), serial passaging of *S. platensis* in increasingly hypersaline media over 5 months yielded strains with improved photosynthetic capacity and salt tolerance, highlighting the potential for genetic adaptation over time.

In summary, we interpret salinity‐induced membrane remodelling as part of an integrated metabolic response to environmental stress, one that reflects energy constraints, phosphorus economy and the biochemical flexibility of different cyanobacterial taxa.

### 4.4 Ecological and Biotechnological Implications

The observed membrane remodelling and changes in surface functional composition under salinity stress have broader implications for both natural ecosystems and biotechnological applications. In aquatic systems experiencing increasing salinity, whether through climate change, sea‐level rise or anthropogenic inputs such as irrigation return flows and road salts (Cheng et al. 2020), cyanobacterial community composition and function will be significantly altered (Kirkwood et al. 2008).

Our findings suggest that surface charge regulation and phosphorus utilisation strategies diverge between marine and non‐marine cyanobacteria, with consequences for ecological interactions. For example, strains like *Syn. PCC6803* and *S. platensis* exhibit increased surface charge and decreased phosphoryl content under saline and hypersaline conditions, which may impair their ability to sorb cations and trace metals (Zhu and Dittrich 2016), initiate biomineralisation reactions (Liang et al. 2013) or remove phosphate from the water column, which are critical processes in regulating eutrophication (Abdoli et al. 2024). In contrast, marine strain *Syn*. PCC8806 maintained or enhanced its phosphoryl content and surface charge, suggesting better resilience and sustained ion exchange potential across salinity gradients. Differences in ion binding dynamics are likely to be most pronounced in freshwater and alkaline systems, where ion concentrations and buffering capacities differ markedly from marine conditions.

Salinity acclimatisation also carries metabolic costs. The energetic demands of Na^+^/H^+^ antiport activity (Gimmler 2000), antioxidant production (Yang et al. 2024) and protein biosynthesis (Klähn et al. 2021) may slow cellular growth rates (Joset et al. 1996) and intensify interspecies competition (Carmona et al. 2021). The salt‐stress pressures could shift microbial community composition towards salt‐tolerant strains that optimise phosphorus usage and exhibit flexible surface structures, particularly, in phosphorus‐limited environments (Giordano and Beardall 2009; Paulo et al. 2018). In this context, the proposed ‘phosphorus engine’ model offers a valuable conceptual framework to explain surface remodelling patterns observed in non‐marine strains.

In the context of eutrophication and bloom control, the altered surface properties observed in this study may influence bloom initiation and toxicity. Bloom‐forming taxa often depend on rapid uptake of phosphorus and nitrogen; if salinity stress reduces this uptake efficiency in some strains while enhancing it in others, community structure and bloom dynamics could shift accordingly (Wagner and Adrian 2009). For example, non‐marine strains with reduced phosphoryl content may be less competitive in eutrophic but saline‐influenced systems, whereas strains like *Syn*. PCC8806, which retain high membrane fluidity and ion uptake potential, may persist or even dominate (Zhang et al. 2016).

From an applied perspective, the biochemical findings inform the selection and optimisation of cyanobacterial strains for biotechnology. In bioremediation systems, for instance, surface charge and functional group availability determine biosorption efficiency and pollutant binding (Wagatsuma 2017). Strains that retain negative surface charge and high phosphoryl density under salinity stress, like *Syn*. PCC8806, may prove advantageous in saline wastewater treatment or brackish aquaculture contexts. Likewise, understanding the lipid remodelling dynamics under stress can support strain engineering for biofilm formation (Loustau et al. 2021), microbial‐induced calcite precipitation (Zhu and Dittrich 2016) or incorporation into cementitious biomaterials (Aceituno‐Caicedo et al. 2023).

Overall, the integration of biophysical surface analyses with physiological observations allows for a deeper understanding of how salinity stress reshapes microbial functionality. As environments become increasingly saline, the ability of cyanobacteria to restructure their membranes, redistribute resources and maintain surface activity will be central to their ecological resilience and industrial utility.

## 5 Conclusion

Our study investigated how salinity stress alters the surface chemistry of three cyanobacterial strains of varying salt tolerance, *Synechocystis* sp. PCC6803, *Synechococcus* sp. PCC8806 and *S. platensis*, using PT, ATR‐FTIR and XPS. Our results revealed that all strains exhibited measurable membrane remodelling under salt stress, but the degree and nature of surface remodelling varied by ecological origin. *Syn*. PCC6803 and *S. platensis* displayed decreased phosphoryl signals, increased ester and polysaccharide signatures and more positive surface charge at higher salinities, suggesting a reduction in phospholipid content and a shift towards more saturated membrane lipids. In contrast, *Syn*. PCC8806 maintained a consistently negative surface charge and exhibited increased phosphoryl abundance under both hypo‐ and hypersaline treatments, suggesting enrichment in anionic phospholipids, possibly PG. We interpret the difference in surface remodelling as evidence of distinct long‐term acclimatisation strategies. The non‐marine strains appear to conserve internal phosphorus by replacing phospholipids with non‐phosphorus‐containing lipids, potentially reallocating resources towards stress‐response processes. The marine strain, by contrast, shows surface‐level remodelling while maintaining functional membrane composition, suggesting a higher inherent salinity resilience. The observations of chemical shifts under salt acclimatisation demonstrate that membrane remodelling alters surface functional groups and macromolecular composition in response to salinity stress, affecting nutrient binding capacity and surface charge. Changes to surface dynamics have downstream impacts on ecological interactions, including nutrient uptake, competition and bloom dynamics, while informing strain selection for applications in bioremediation, biosorption and industrial cultivation in saline environments.

## Author Contributions

DA‐C and MD conceptualized, developed, and designed the study. DA‐C executed methodology and laboratory experiments with contributions from NK and YZ. DA‐C analyzed data and results with support and feedback from MD. DA‐C wrote the first draft of the manuscript and prepared visuals, with MD contributing revisions and edits. MD acquired funding and provided supervision and project administration.

## Conflicts of Interest

The authors declare no conflicts of interest.

## Supporting information


**Table S1:** Compositions for regular and modified BG‐11, ASN‐III and Zarrouk's media. Media were autoclaved at 120°C for 20 min. Zarrouk's medium was prepared in two parts and mixed after autoclaving.
**Figure S1:** Growth curves for (a) *Syn*. PCC6803 under freshwater (P68‐F) and saline (P68‐S) conditions and (b) *Spirulina platensis* for freshwater (SP‐F), saline (SP‐S) and hypersaline (SP‐H) conditions up to 30 days of growth. Curves were generated using Gompertz linear modelling on Origin. Data for *Syn*. PCC8806 not shown.
**Figure S2:** Potentiometric titration charge excess models created using LPM under treatments under freshwater (–F), saline (–S) and hypersaline (–H) conditions for (a) P68, (b) P88 and (c) SP cultures. The pH value where the charge excess is equal to 0 is known as the point of zero charge (PZC) and represents equal abundance of negatively charged acidic sites and positively charged basic sites on functional groups. Higher abundance of more acidic sites results in a more negative charge, while more basic sites contribute to a more positive charge. P68 and SP results show more positively charged surfaces and a lower PZC at higher salinity conditions. P88 groups show similar PZC across salinity conditions and increasing abundance of acidic groups under both freshwater and hypersaline conditions.
**Figure S3:** PCA of functional group distribution across pH of the background (B), P68‐F and P68‐S treatments for (a) carboxyl groups, (b) phosphoryl groups, (c) amine groups and (d) hydroxyl groups. Clustering of pKa values (blue) around treatments (red) identifies the pKa ranges where the functional group abundance changed the most. The shifts in values are consistent with remodelling of functional groups in the cell surface architecture.
**Table S2:** Percent variability of the original dataset represented by each extracted principal component and the standardised score data for each of the functional groups for P68.
**Table S3:** Extracted eigenvectors for each pH factor for PC1 and PC2 for the P68 pKa distribution analysis. Loadings with an absolute value greater than 0.35 are considered large (DiStefano et al. 2009).
**Figure S4:** PCA of functional group distribution across pH of the background (B), P88‐F, P88‐S, P88‐H and treatments for (a) carboxyl groups, (b) phosphoryl groups, (c) amine groups and (d) hydroxyl groups. Clustering of pKa values (blue) around treatments (red) identifies the pKa ranges where the functional group abundance changed the most. The shifts in values are consistent with remodelling of functional groups in the cell surface architecture.
**Table S4:** Percent variability of the original dataset represented by each extracted principal component and the standardised score data for each of the functional groups for P88.
**Table S5:** Extracted eigenvectors for each pH factor for PC1 and PC2 for the P88 pKa distribution analysis. Loadings with an absolute value greater than 0.35 are considered large (DiStefano et al. 2009).
**Figure S5:** PCA of functional group distribution across pH of the background (B), SP‐F, SP‐S and SP‐H treatments for (a) carboxyl groups, (b) phosphoryl groups, (c) amine groups and (d) hydroxyl groups. Clustering of pKa values (blue) around treatments (red) identifies the pKa ranges where the functional group abundance changed the most. The shifts in values are consistent with remodelling of functional groups in the cell surface architecture.
**Table S6:** Percent variability of the original dataset represented by each extracted principal component and the standardised score data for each of the functional groups for SP.
**Table S7:** Extracted eigenvectors for each pH factor for PC1 and PC2 for the SP pKa distribution analysis. Loadings with an absolute value greater than 0.35 are considered large (DiStefano et al. 2009).
**Figure S6:** Averaged FTIR low‐frequency fingerprint region spectra (*n* = 3) for P68 experiments under native freshwater (–F) and saline (–S) conditions. Key spectral regions are labelled and include ~1740 cm^−1^ (*ν*C═O of esters), ~1650 cm^−1^ (amide I), ~1540 cm^−1^ (amide II), ~1455–1300 cm^−1^ (amide III), 1388 cm^−1^ (*δ*_*ac*_CH2/*δ*_*ac*_CH3 and *ν*_*s*_COO^−^), ~1149–915 cm^−1^ (carbohydrates and polysaccharides), ~1078–915 cm^−1^ (phosphates and carbohydrates) and < 915 cm^−1^ (–PO, –SO and aromatic –CH). The P68‐F and P88‐S treatments differ noticeably in ester, phosphoryl and carbohydrate vibration bands.
**Figure S7:** Averaged FTIR low‐frequency fingerprint region spectra (*n* = 3) for P88 experiments under freshwater (–F), native saline (–S) and hypersaline (–H) conditions. Key spectral regions are labelled and include ~1740 cm^−1^ (*ν*C═O of esters), ~1650 cm^−1^ (amide I), ~1540 cm^−1^ (amide II), ~1455–1300 cm^−1^ (amide III), 1388 cm^−1^ (*δ*_*ac*_CH2/*δ*_*ac*_CH3 and *ν*_*s*_COO^−^), ~1149–915 cm^−1^ (carbohydrates and polysaccharides), ~1078–915 cm^−1^ (phosphates and carbohydrates) and < 915 cm^−1^ (–PO, –SO and aromatic –CH). The P88‐F, P88‐S and P88‐H treatments differ noticeably in the ester, phosphoryl and carbohydrate regions.
**Figure S8:** Averaged FTIR low‐frequency fingerprint region spectra (*n* = 3) for SP experiments under native freshwater (–F), saline (–S) and hypersaline (–H) conditions. Key spectral regions are labelled and include ~1740 cm^−1^ (*ν*C═O of esters), ~1650 cm^−1^ (amide I), ~1540 cm^−1^ (amide II), ~1455–1300 cm^−1^ (amide III), 1388 cm^−1^ (*δ*_*ac*_CH2/*δ*_*ac*_CH3 and ν_*s*_COO^−^), ~1149–915 cm^−1^ (carbohydrates and polysaccharides), ~1078–915 cm^−1^ (phosphates and carbohydrates) and < 915 cm^−1^ (–PO, –SO and aromatic –CH). The SP‐F, SP‐S and SP‐H treatments differ noticeably in the ester, phosphoryl and carbohydrate regions.
**Table S8:** Peaks, calculated areas and *R*_3/2_ values for P68, P88 and SP treatments. Larger *R*_3/2_ values are interpreted as less saturated, more branched lipid profiles, while lower values correspond to more saturated and less branched lipids. More branching and less saturation are consistent to more fluid membranes, while more saturation and less branching are linked to less fluid membranes.
**Figure S9:** Averaged FTIR high‐frequency lipid region spectra (*n* = 3) P68 experiments. Regions are labelled and correspond to hydroxyl and water‐related spectral vibrations, or possible triple bond and metal‐related bond vibrations. Key regions of interest are the –CH group vibrations in the ~2970–2855 cm^−1^ range that represent vibrations of alkanes, alkenes and fatty acids.
**Figure S10:** Spectra deconvolution for the hydrocarbon region of the (a) P68‐F and (b) P68‐S treatments. Deconvolution separated both the *ν*_as_ and *ν*_s_ vibration bands of methyl and methylene, of which the proportional area under the peak of *ν*_as_ were used to calculate the *R*_3/2_ ratio.
**Figure S11:** Averaged FTIR high‐frequency lipid region spectra (*n* = 3) for the P88 experiments. Regions are labelled and correspond to hydroxyl and water‐related spectral vibrations or possible triple bond and metal‐related bond vibrations. Key regions of interest are the –CH group vibrations in the ~2970–2855 cm^−1^ range that represent vibrations of alkanes, alkenes and fatty acids.
**Figure S12:** Spectra deconvolution for the hydrocarbon region of the (a) P88‐F, (b) P88‐S and (c) P88‐H treatments. Deconvolution separated both the *ν*_as_ and *ν*_s_ vibration bands of methyl and methylene, of which the proportional area under the peak of *ν*_as_ were used to calculate the *R*_3/2_ ratio.
**Figure S13:** Averaged FTIR high‐frequency lipid region spectra (*n* = 3) for the SP experiments. Regions are labelled and correspond to hydroxyl and water‐related spectral vibrations or possible triple bond and metal‐related bond vibrations. Key regions of interest are the –CH group vibrations in the ~2970–2855 cm^−1^ range that represent vibrations of alkanes, alkenes and fatty acids.
**Figure S14:** Spectra deconvolution for the hydrocarbon region of the (a) SP‐F, (b) SP‐S and (c) SP‐H treatments. Deconvolution separated both the *ν*_as_ and *ν*_s_ vibration bands of methyl and methylene, of which the proportional area under the peak of *ν*_as_ were used to calculate the *R*_3/2_ ratio.

## Data Availability

The data that support the findings of this study are available on request from the corresponding author. The data are not publicly available due to privacy or ethical restrictions.
