# Supplementary material for: Impact of Salinity on Cell Surface Chemistry of Cyanobacteria From Freshwater, Marine and Alkaline Environments: A Hidden Phosphorus Engine
Source: Environ Microbiol. 2025 Sep 30;27(10):e70180. doi: 10.1111/1462-2920.70180 (PMC12482097; doi:10.1111/1462-2920.70180)
Supplement: Supplementary file 1 — Table S1: Compositions for regular and modified BG‐11, ASN‐III and Zarrouk's media. Media were autoclaved at 120°C for 20 min. Zarrouk's medium was prepared in two parts and mixed after autoclaving. Figure S1: Growth curves for (a) Syn. PCC6803 under freshwater (P68‐F) and saline (P68‐S) conditions and (b) Spirulina platensis for freshwater (SP‐F), saline (SP‐S) and hypersaline (SP‐H) conditions up to 30 days of growth. Curves were generated using Gompertz linear modelling on Origin. Data for Syn. PCC8806 not shown. Figure S2: Potentiometric titration charge excess models created using LPM under treatments under freshwater (–F), saline (–S) and hypersaline (–H) conditions for (a) P68, (b) P88 and (c) SP cultures. The pH value where the charge excess is equal to 0 is known as the point of zero charge (PZC) and represents equal abundance of negatively charged acidic sites and positively charged basic sites on functional groups. Higher abundance of more acidic sites results in a more negative charge, while more basic sites contribute to a more positive charge. P68 and SP results show more positively charged surfaces and a lower PZC at higher salinity conditions. P88 groups show similar PZC across salinity conditions and increasing abundance of acidic groups under both freshwater and hypersaline conditions. Figure S3: PCA of functional group distribution across pH of the background (B), P68‐F and P68‐S treatments for (a) carboxyl groups, (b) phosphoryl groups, (c) amine groups and (d) hydroxyl groups. Clustering of pKa values (blue) around treatments (red) identifies the pKa ranges where the functional group abundance changed the most. The shifts in values are consistent with remodelling of functional groups in the cell surface architecture. Table S2: Percent variability of the original dataset represented by each extracted principal component and the standardised score data for each of the functional groups for P68. Table S3: Extracted eigenvectors for each pH factor f [file EMI-27-e70180-s001.docx]

# **Supplementary Information**

# **1. Methods**

### **Bacteria culturing and growth curves**

#### **Medium compositions**

Table S1: Compositions for regular and modified BG-11, ASN-III, and Zarrouk’s media. Media were autoclaved at 120°C for 20 min. Zarrouk’s medium was prepared in two parts and mixed after autoclaving.

| Medium | Base Composition (per liter) | | Treatment | Modifications (per liter) |
| --- | --- | --- | --- | --- |
| BG-11 | Autoclaved  6.0 mg citric acid  1.0 mg EDTA disodium salt  75.0 mg MgSO_4_·7H_2_O  40.0 mg K_2_HPO_4_·3H_2_O  1,500.0 mg NaNO_3_ | Filter-sterilized  36.0 mg CaCl_2_∙2H_2_O  6.0 mg ammonium ferric citrate  20.0 mg Na_2_CO_3_  1 mL A5 Micronutrients | P68-F  “Freshwater” | n/a |
|  |  |  | P68-S  “Saline” | + 28,390 mg NaCl |
| ASN-III | Autoclaved  3.0 mg citric acid  0.5 mg EDTA disodium salt  25,000 mg NaCI  3,500 mg MgSO_4_·7H_2_O,  2,000 mg MgCI_2_·6H_2_O  750 mg NaNO_3_  750 mg K_2_HPO_3_·3H_2_O  500 mg KCI | Filter-sterilized  6.0 mg ammonium ferric citrate  36.0 mg CaCl2∙2H2O  20.0 mg NaCO3,  1 mL A5 Micronutrients | P88-F  “Freshwater” | -24,500 mg NaCl |
|  |  |  | P88-S  “Saline” | n/a |
|  |  |  | P88-H  “Freshwater” | +20,000 mg NaCl |
| Zarrouk’s | Part 1  16,800 mg NaHCO_3_  1,000 mg K_2_HPO_4_·3H_2_O  Part 2  2,500 mg NaNO_3_  1,000 mg K_2_SO_4_  1,000 mg NaCI  200 mg MgSO_4_·7H_2_O  40 mg CaCI2  10 mg FeSO_4_·7H_2_O  80 mg EDTA disodium salt | Filter-Sterilized  1 mL A5 Micronutrients | SP-F  “Saline” | n/a |
|  |  |  | SP-S  “Freshwater” | + 20,390 mg NaCl |
|  |  |  | SP-H  “Saline” | + 50,390 mg NaCl |
| A5 Micro-nutrients | 2.9 mg H_3_BO_3_,  1.8 mg MnCl_2_·4H_2_O,  0.22 mg ZnSO_4_·7H_2_O  0.39 mg NaMoO4·2H_2_O,  0.079 mg CuSO_4_·5H_2_O  0.049 mg Co(NO_3_)_2_·6H_2_O | | | |

#### **Growth Curves**


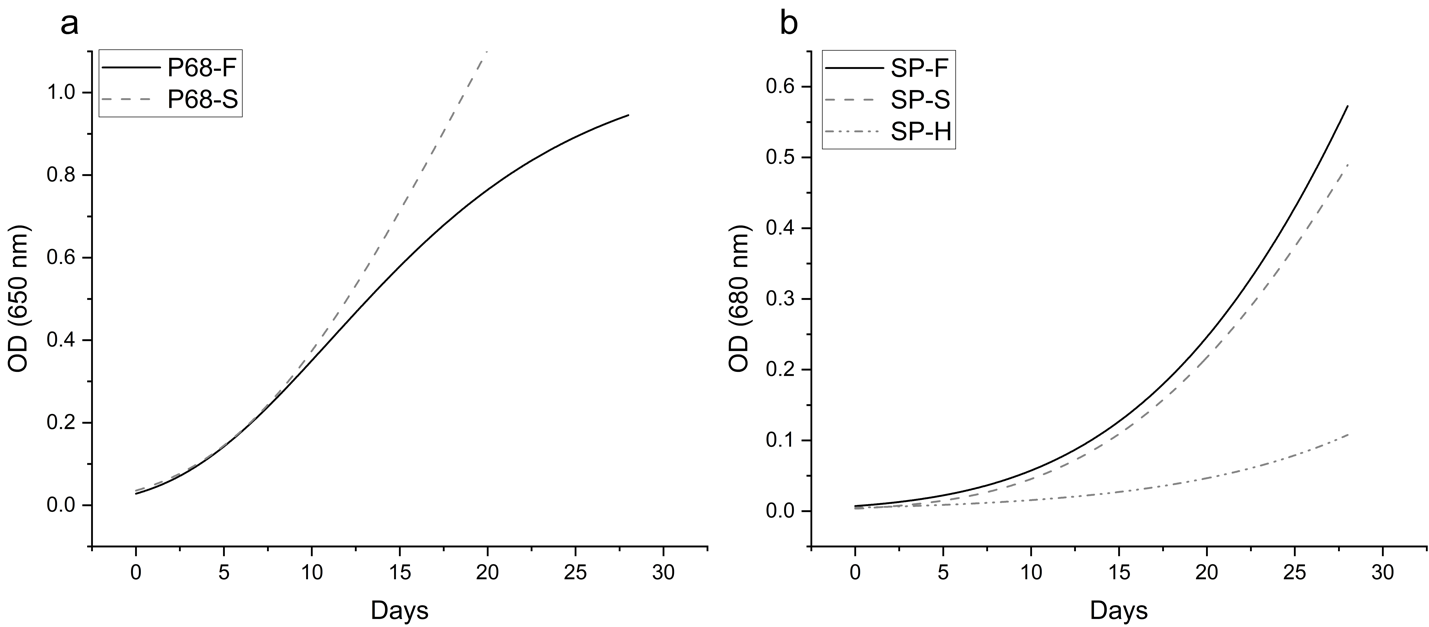


Figure S1: Growth curves for (a) *Syn.* PCC6803 under freshwater (P68-F) and saline (P68-S) conditions, and (b) *S. platensis* for freshwater (SP-F), saline (SP-S), and hypersaline (SP-H) conditions up to 30 days of growth. Curves were generated using Gompertz linear modelling on Origin. Data for *Syn.* PCC8806 not shown.

#### **Potentiometric Titration**

Surface site concentrations were determined using duplicate dynamic endpoint acid-base titrations of the cyanobacterial suspensions in the background 0.1 M NaNO_3_ electrolyte solution. The dynamic endpoint titration program added variable amounts of titrant, with increasingly smaller volumes added at the slope of the curve. Titrations were performed in a glass vessel with a lid using a Metrohm Titrando 905 auto-titrator interfaced by Titrino software Tiamo 2.2 to a computer. Separate burette exchange units were used for the acid and base. Prior to titrations, the pH electrode was three-point calibrated with pH buffers (pH 4, 7, and 10).

The bacterial titrations were performed using 3.0 mL of the resuspended cells in the glass vessel with a magnetic stir bar. The samples were then slowly acidified to pH 3, then titrated to pH 10, and repeated once more. Each sample was analyzed in duplicate.  The titration data was expressed in the form of charge excess per gram, which was calculated by

$\frac{C_{bj}-C_{aj}}{0.009 g}= \sum_{i=1}^{n} \left( \frac{K_{ai}L_{Ti}}{K_{ai}+[H^{+}]_{j}} \right)+S$ (eq 1)

Where C_aj_ and C_bj_ are the total concentrations of acid and base at each j addition of titrant and 0.009 g is the mass of dry cell weight in each sample. The surface binding sites were calculated based on charge excess and the functional group determination was performed using a linear programming method (LPM) over a pH range of 4- 10 at 0.2 unit intervals represented by the right side of the equation (Cox *et al.*, 1999; Dittrich and Sibler, 2005, 2006). The pKa ranges to determine the average concentrations of each functional group were based on previous works (Dittrich and Sibler, 2005; Diloreto *et al.*, 2021). Total concentrations of surface binding sites were determined using the sum of the number (n) of the monoprotic ligands (L_Ti_) and the constant (S) acid neutralization capacity of the cell surfaces. The charge excess models developed were plotted against the titration data and used to compare charge excess differences between treatments.

## **Results**

### **Potentiometric Titration**

#### **Charge Excess Models**


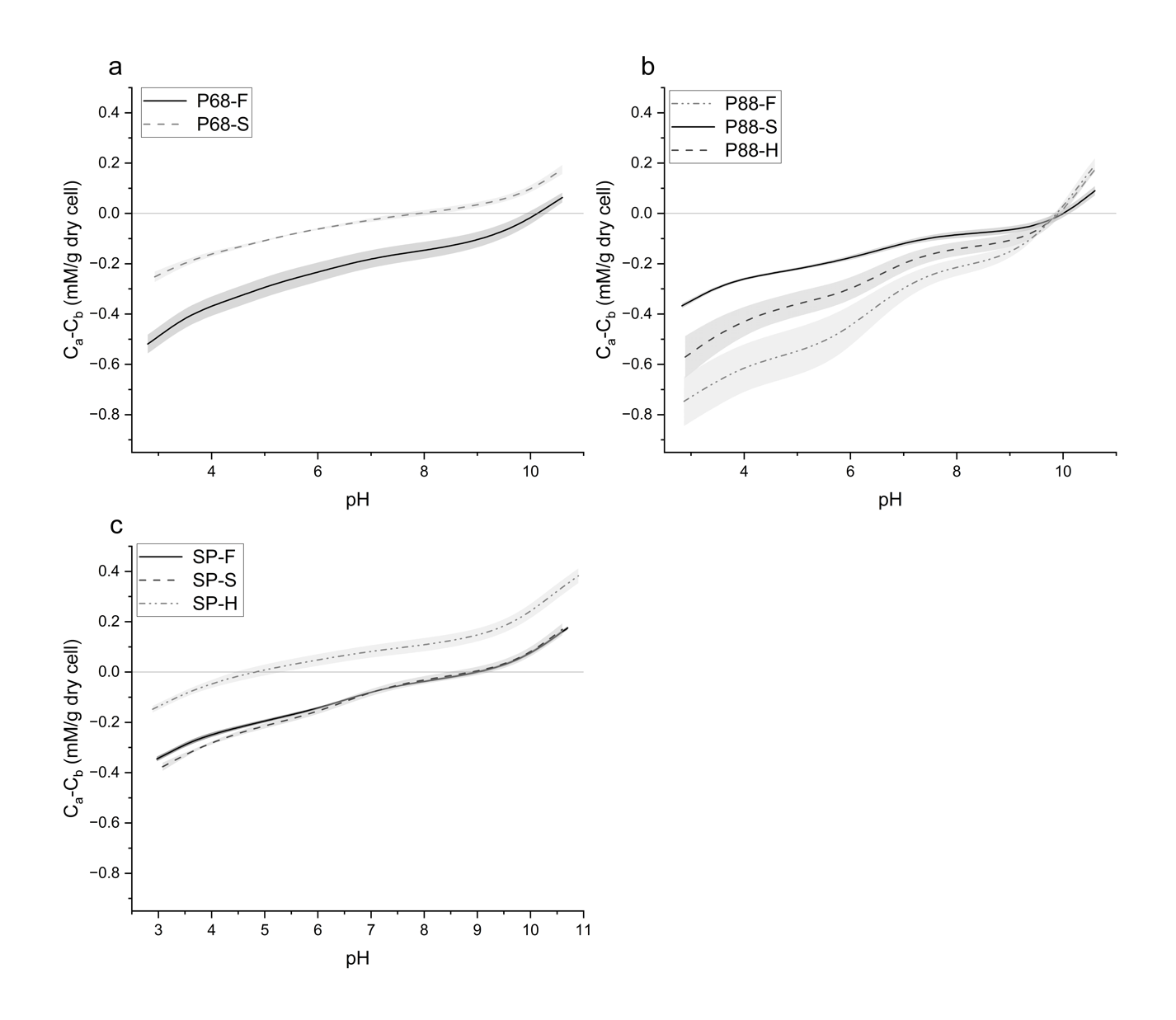


Figure S2: Potentiometric titration charge excess models created using LPM under treatments under freshwater (-F), saline (-S), and hypersaline (-H) conditions for (a) P68, (b) P88, and (c) SP cultures. The pH value where the charge excess is equal to 0 is known as the Point of Zero Charge (PZC) and represents equal abundance of negatively charged acidic sites and positively charged basic sites on functional groups. Higher abundance of more acidic sites results in a more negative charge, while more basic sites contribute to a more positive charge. P68 and SP results show more positively charged surfaces, and a lower PZC at higher salinity conditions. P88 groups show similar PZC across salinity conditions, and increasing abundance of acidic groups under both freshwater and hypersaline conditions.


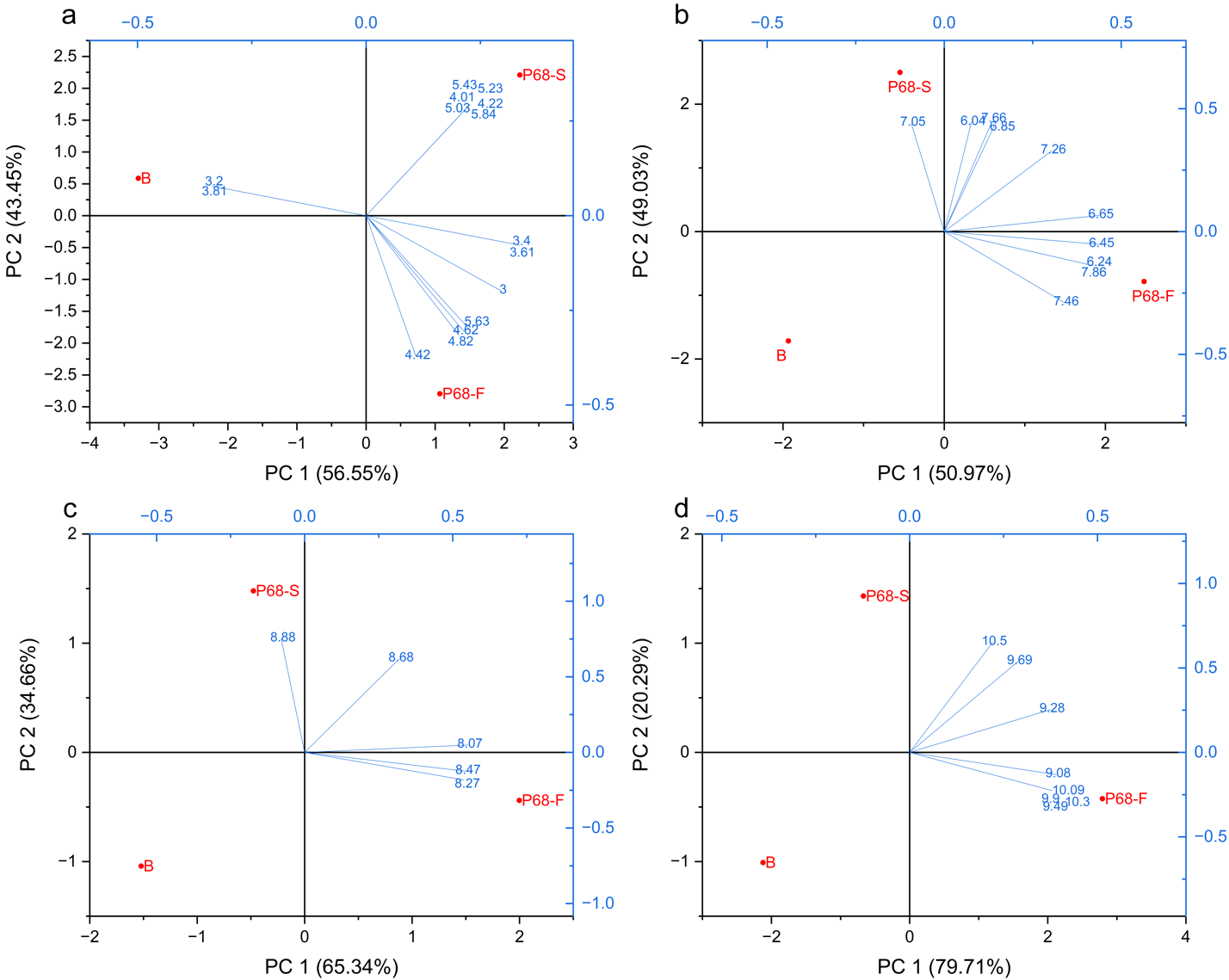


Figure S3: PCA of functional group distribution across pH of the background (B), P68-F and P68-S treatments for (a) carboxyl groups, (b) phosphoryl groups, (c) amine groups and (d) hydroxyl groups. Clustering of pKa values (blue) around treatments (red) identifies the pKa ranges where the functional group abundance changed the most. The shifts in values are consistent with remodelling of functional groups in the cell surface architecture.

Table S2: Percent variability of the original dataset represented by each extracted principal component and the standardized score data for each of the functional groups for P68.

|  | Carboxyl | | Phosphoryl | | Amine | | Hydroxyl | |
| --- | --- | --- | --- | --- | --- | --- | --- | --- |
|  | PC1 | PC2 | PC1 | PC2 | PC1 | PC2 | PC1 | PC2 |
| Variance | 56.55% | 43.45% | 65.34% | 34.66% | 79.71% | 20.29% | 48.26% | 29.07% |
| Standardized Scores | | | | | | | | |
| B | -3.30 | 0.59 | -1.52 | -1.04 | -2.12 | -1.01 | -2.67 | -2.24 |
| P68-F | 1.07 | -2.79 | 2.00 | -0.44 | 2.79 | -0.42 | 0.93 | 1.63 |
| P68-S | 2.23 | 2.21 | -0.48 | 1.48 | -0.67 | 1.43 | -1.60 | 1.93 |

Table S3: Extracted eigenvectors for each pH factor for PC1 and PC2 for the P68 pKa distribution analysis. Loadings with an absolute value greater than 0.35 are considered large (DiStefano *et al.*, 2009)

| Carboxyl | | | Phosphoryl | | | Amine | | | Hydroxyl | | |
| --- | --- | --- | --- | --- | --- | --- | --- | --- | --- | --- | --- |
| pH | PC1 | PC2 | pH | PC1 | PC2 | pH | PC1 | PC2 | pH | PC1 | PC2 |
| 3 | **0.30** | -0.20 | 6.04 | 0.08 | **0.44** | 8.07 | **0.55** | 0.05 | 9.08 | **0.39** | -0.13 |
| 3.2 | **-0.34** | 0.08 | 6.24 | **0.42** | -0.14 | 8.27 | **0.54** | -0.18 | 9.28 | **0.37** | 0.25 |
| 3.4 | **0.34** | -0.08 | 6.45 | **0.44** | -0.05 | 8.47 | **0.55** | -0.12 | 9.49 | **0.38** | -0.23 |
| 3.61 | **0.34** | -0.08 | 6.65 | **0.44** | 0.07 | 8.68 | 0.32 | **0.62** | 9.69 | 0.29 | **0.54** |
| 3.81 | **-0.34** | 0.08 | 6.82 | 0.14 | **0.43** | 8.88 | -0.08 | **0.75** | 9.9 | **0.38** | -0.23 |
| 4.01 | 0.23 | 0.29 | 7.05 | -0.09 | **0.44** |  |  |  | 10.09 | **0.38** | -0.23 |
| 4.22 | 0.23 | 0.29 | 7.26 | 0.30 | 0.33 |  |  |  | 10.3 | **0.38** | -0.23 |
| 4.42 | 0.11 | **-0.37** | 7.46 | 0.34 | -0.29 |  |  |  | 10.5 | 0.22 | **0.65** |
| 4.62 | 0.21 | **-0.31** | 7.66 | 0.13 | **0.43** |  |  |  |  |  |  |
| 4.82 | 0.20 | **-0.32** | 7.86 | **0.42** | -0.14 |  |  |  |  |  |  |
| 5.03 | 0.23 | 0.29 |  |  |  |  |  |  |  |  |  |
| 5.23 | 0.23 | 0.29 |  |  |  |  |  |  |  |  |  |
| 5.43 | 0.23 | 0.29 |  |  |  |  |  |  |  |  |  |
| 5.63 | 0.22 | **-0.30** |  |  |  |  |  |  |  |  |  |
| 5.84 | 0.23 | 0.29 |  |  |  |  |  |  |  |  |  |


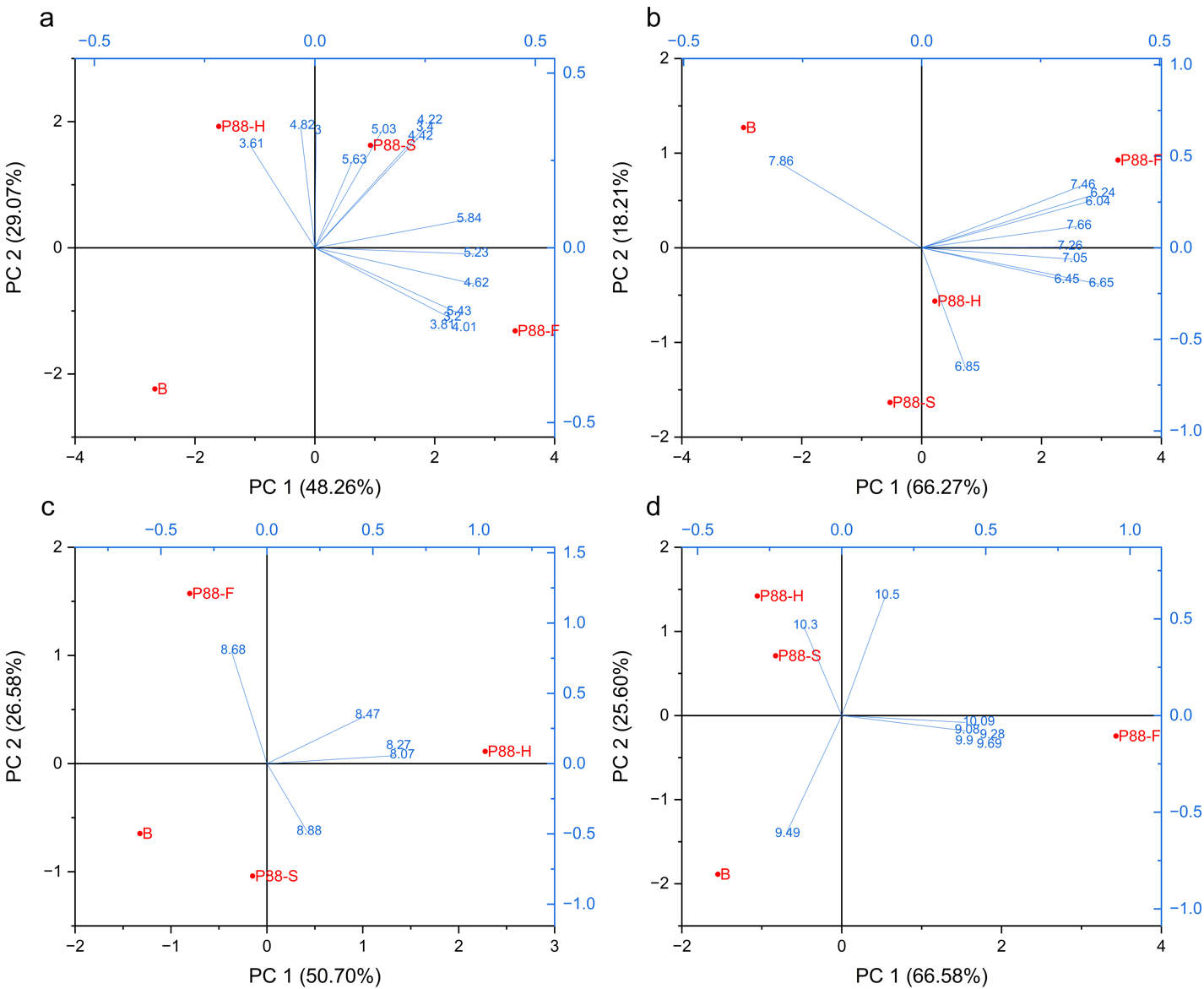


Figure S4: PCA of functional group distribution across pH of the background (B), P88-F, P88-S, P88-H and treatments for (a) carboxyl groups, (b) phosphoryl groups, (c) amine groups and (d) hydroxyl groups. Clustering of pKa values (blue) around treatments (red) identifies the pKa ranges where the functional group abundance changed the most. The shifts in values are consistent with remodelling of functional groups in the cell surface architecture

Table S4: Percent variability of the original dataset represented by each extracted principal component and the standardized score data for each of the functional groups for P88.

|  | Carboxyl | | Phosphoryl | | Amine | | Hydroxyl | |
| --- | --- | --- | --- | --- | --- | --- | --- | --- |
|  | PC1 | PC2 | PC1 | PC2 | PC1 | PC2 | PC1 | PC2 |
| Variance | 48.26% | 29.07% | 66.27% | 18.21% | 50.70% | 26.58% | 66.58% | 25.60% |
| Standardized Scores | | | | | | | | |
| B | -2.67 | -2.24 | -2.97 | 1.27 | -1.32 | -0.65 | -1.55 | -1.89 |
| P88-F | 3.34 | -1.32 | 3.28 | 0.93 | -0.81 | 1.57 | 3.43 | -0.24 |
| P88-S | 0.93 | 1.63 | -0.53 | -1.63 | -0.15 | -1.04 | -0.83 | 0.71 |
| P88-H | -1.60 | 1.93 | 0.22 | -0.56 | 2.28 | 0.11 | -1.06 | 1.42 |

Table S5: Extracted eigenvectors for each pH factor for PC1 and PC2 for the P88 pKa distribution analysis. Loadings with an absolute value greater than 0.35 are considered large (DiStefano *et al.*, 2009).

| Carboxyl | | | Phosphoryl | | | Amine | | | Hydroxyl | | |
| --- | --- | --- | --- | --- | --- | --- | --- | --- | --- | --- | --- |
| pH | PC1 | PC2 | pH | PC1 | PC2 | pH | PC1 | PC2 | pH | PC1 | PC2 |
| 3 | 0.00 | 0.33 | 6.04 | **0.36** | 0.26 | 8.07 | **0.60** | 0.06 | 9.08 | **0.43** | -0.08 |
| 3.2 | 0.31 | -0.20 | 6.24 | **0.36** | 0.29 | 8.27 | **0.60** | 0.06 | 9.28 | **0.43** | -0.08 |
| 3.4 | 0.25 | 0.34 | 6.45 | 0.30 | -0.18 | 8.47 | **0.47** | 0.34 | 9.49 | -0.19 | **-0.61** |
| 3.61 | -0.15 | 0.29 | 6.65 | **0.37** | -0.20 | 8.68 | -0.17 | **0.80** | 9.69 | **0.43** | -0.08 |
| 3.81 | 0.31 | -0.20 | 6.82 | 0.09 | **-0.66** | 8.88 | 0.19 | **-0.49** | 9.9 | **0.43** | -0.08 |
| 4.01 | 0.31 | -0.20 | 7.05 | 0.32 | -0.06 |  |  |  | 10.09 | **0.43** | 0.04 |
| 4.22 | 0.25 | 0.34 | 7.26 | 0.31 | 0.00 |  |  |  | 10.3 | -0.13 | **0.46** |
| 4.42 | 0.24 | 0.32 | 7.46 | 0.34 | 0.34 |  |  |  | 10.5 | 0.15 | **0.62** |
| 4.62 | **0.36** | -0.10 | 7.66 | 0.33 | 0.12 |  |  |  |  |  |  |
| 4.82 | -0.03 | **0.35** | 7.86 | -0.30 | **0.47** |  |  |  |  |  |  |
| 5.03 | 0.15 | 0.34 |  |  |  |  |  |  |  |  |  |
| 5.23 | **0.36** | -0.02 |  |  |  |  |  |  |  |  |  |
| 5.43 | 0.32 | -0.19 |  |  |  |  |  |  |  |  |  |
| 5.63 | 0.09 | 0.25 |  |  |  |  |  |  |  |  |  |
| 5.84 | **0.35** | 0.08 |  |  |  |  |  |  |  |  |  |


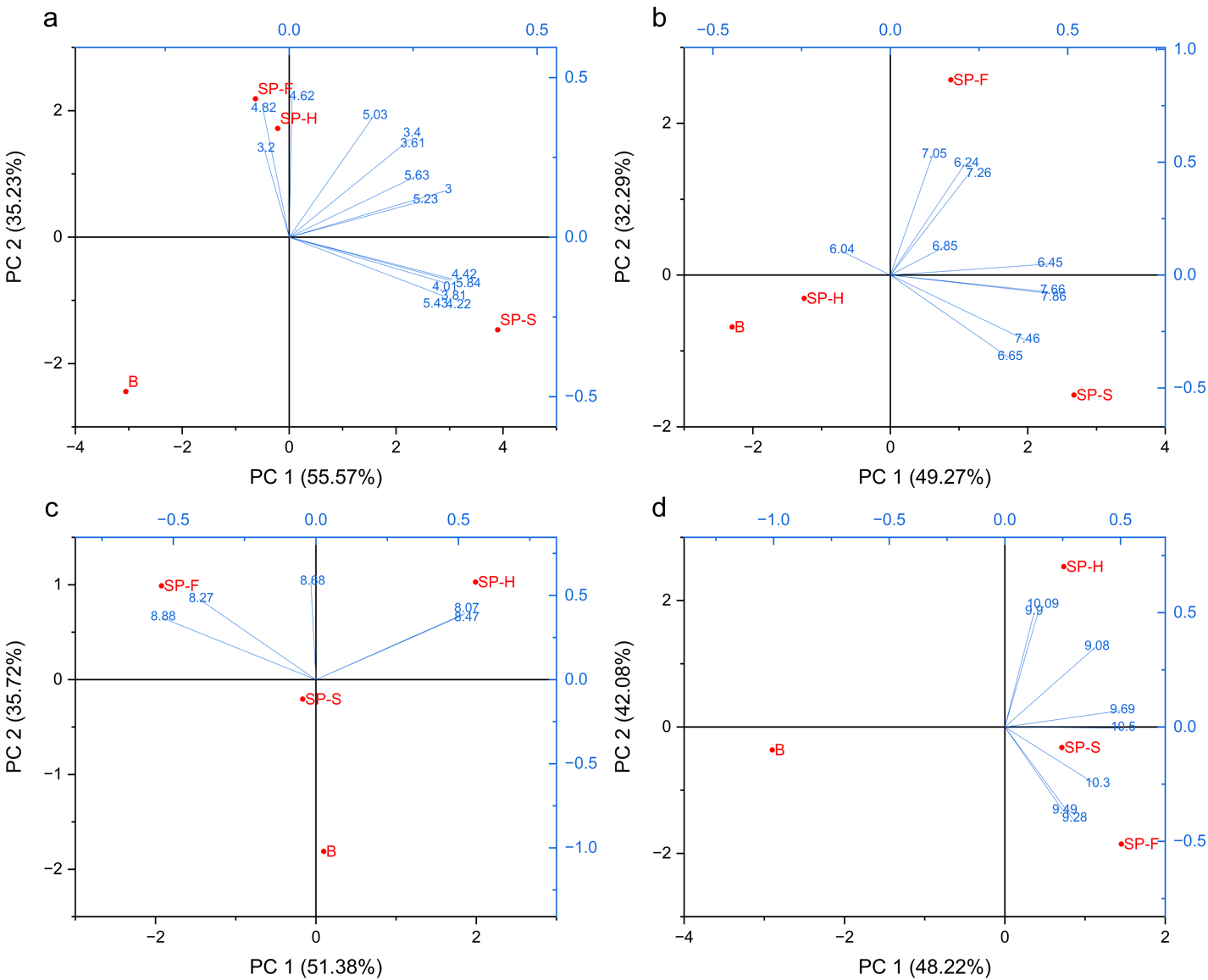


Figure S5: PCA of functional group distribution across pH of the background (B), SP-F, SP-S and SP-H treatments for (a) carboxyl groups, (b) phosphoryl groups, (c) amine groups and (d) hydroxyl groups. Clustering of pKa values (blue) around treatments (red) identifies the pKa ranges where the functional group abundance changed the most. The shifts in values are consistent with remodelling of functional groups in the cell surface architecture

Table S6: Percent variability of the original dataset represented by each extracted principal component and the standardized score data for each of the functional groups for SP.

|  | Carboxyl | | Phosphoryl | | Amine | | Hydroxyl | |
| --- | --- | --- | --- | --- | --- | --- | --- | --- |
|  | PC1 | PC2 | PC1 | PC2 | PC1 | PC2 | PC1 | PC2 |
| Variance | 55.57% | 35.23% | 49.27% | 32.29% | 51.38% | 35.72% | 48.22% | 42.08% |
| Standardized Scores | | | | | | | | |
| B | -3.06 | -2.44 | -2.30 | -0.69 | 0.10 | -1.81 | -2.90 | -0.36 |
| SP-F | -0.63 | 2.19 | 0.88 | 2.57 | -1.93 | 0.99 | 1.45 | -1.85 |
| SP-S | 3.90 | -1.46 | 2.67 | -1.58 | -0.16 | -0.21 | 0.71 | -0.32 |
| SP-H | -0.21 | 1.72 | -1.25 | -0.31 | 1.99 | 1.03 | 0.74 | 2.54 |

Table S7: Extracted eigenvectors for each pH factor for PC1 and PC2 for the SP pKa distribution analysis. Loadings with an absolute value greater than 0.35 are considered large (DiStefano *et al.*, 2009).

| Carboxyl | | | Phosphoryl | | | Amine | | | Hydroxyl | | |
| --- | --- | --- | --- | --- | --- | --- | --- | --- | --- | --- | --- |
| pH | PC1 | PC2 | pH | PC1 | PC2 | pH | PC1 | PC2 | pH | PC1 | PC2 |
| 3 | 0.32 | 0.15 | 6.04 | -0.14 | 0.11 | 8.07 | **0.52** | **0.39** | 9.08 | **0.39** | **0.35** |
| 3.2 | -0.05 | 0.28 | 6.24 | 0.21 | **0.49** | 8.27 | **-0.41** | **0.48** | 9.28 | 0.29 | **-0.40** |
| 3.4 | 0.24 | 0.31 | 6.45 | **0.45** | 0.05 | 8.47 | **0.52** | **0.38** | 9.49 | 0.25 | **-0.37** |
| 3.61 | 0.24 | 0.31 | 6.65 | 0.33 | **-0.36** | 8.68 | -0.02 | **0.58** | 9.69 | **0.50** | 0.07 |
| 3.81 | 0.31 | -0.18 | 6.82 | 0.15 | 0.12 | 8.88 | **-0.54** | **0.37** | 9.9 | 0.13 | **0.50** |
| 4.01 | 0.32 | -0.14 | 7.05 | 0.12 | **0.53** |  |  |  | 10.09 | 0.15 | **0.51** |
| 4.22 | 0.31 | -0.18 | 7.26 | 0.23 | **0.47** |  |  |  | 10.3 | **0.39** | -0.25 |
| 4.42 | 0.33 | -0.13 | 7.46 | **0.38** | -0.29 |  |  |  | 10.5 | **0.50** | -0.00 |
| 4.62 | 0.01 | **0.43** | 7.66 | **0.44** | -0.07 |  |  |  |  |  |  |
| 4.82 | -0.05 | **0.42** | 7.86 | **0.44** | -0.08 |  |  |  |  |  |  |
| 5.03 | 0.17 | 0.38 |  |  |  |  |  |  |  |  |  |
| 5.23 | 0.27 | 0.11 |  |  |  |  |  |  |  |  |  |
| 5.43 | 0.31 | -0.18 |  |  |  |  |  |  |  |  |  |
| 5.63 | 0.25 | 0.19 |  |  |  |  |  |  |  |  |  |
| 5.84 | 0.33 | -0.14 |  |  |  |  |  |  |  |  |  |

### **Fourier Transform Infrared Spectroscopy (FTIR)**

#### **Detailed Fingerprint Spectra**


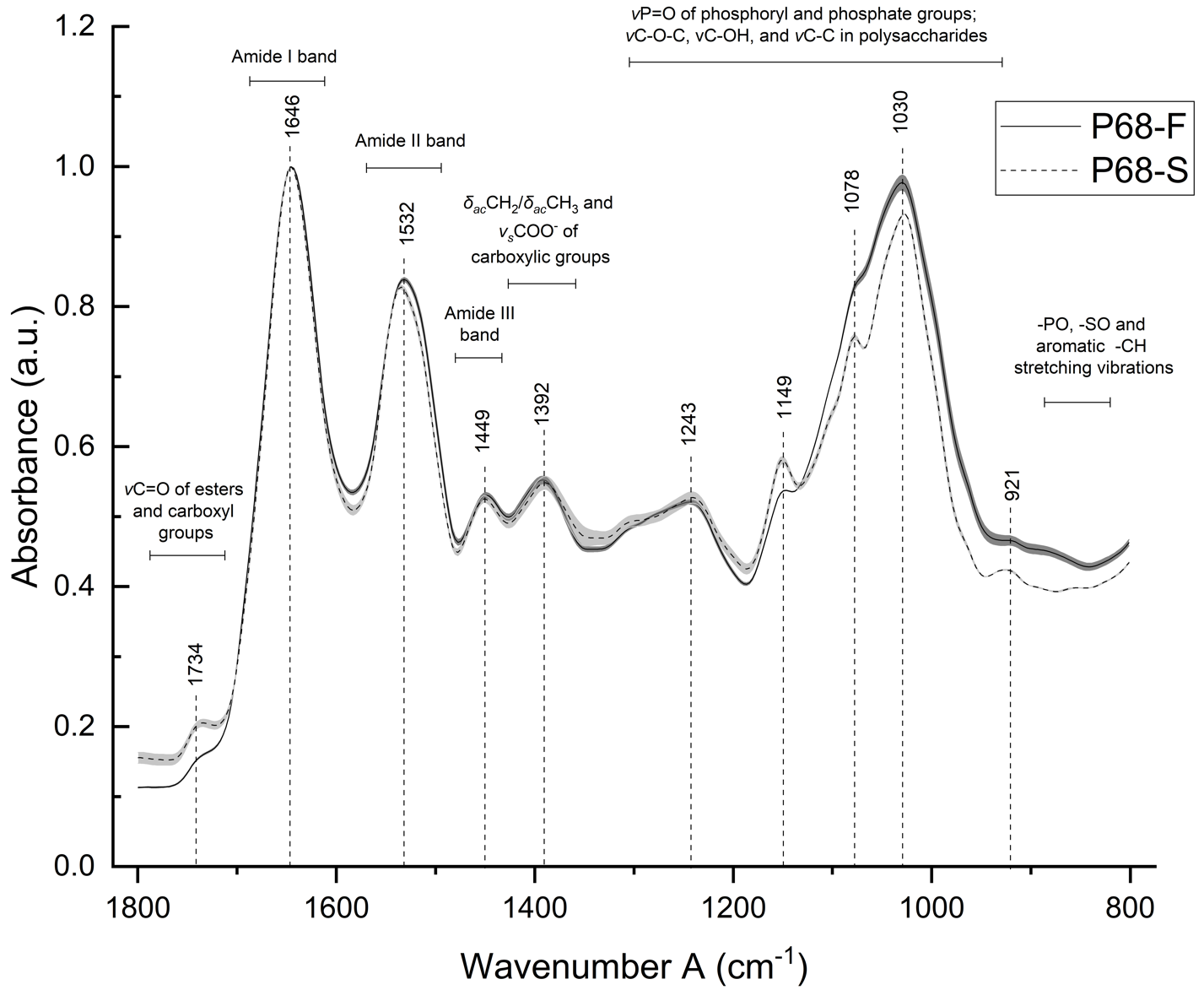


Figure S6: Averaged FTIR low-frequency fingerprint region spectra (n=3) for P68 experiments under native freshwater (-F) and saline (-S) conditions. Key spectral regions are labelled and include ~1740 cm⁻¹ (νC=O of esters), ~1650 cm⁻¹ (amide I), ~1540 cm⁻¹ (amide II), ~1455–1300 cm⁻¹ (amide III), 1388 cm⁻¹ (*δ_ac_*CH2/*δ_ac_*CH3 and ν*_s_*COO^-^), ~1149–915 cm⁻¹ (carbohydrates and polysaccharides), ~1078–915 cm⁻¹ (phosphates and carbohydrates), and < 915 cm⁻¹ (-PO, -SO and aromatic -CH). The P68-F and P88-S treatments differ noticeably in ester, phosphoryl, and carbohydrate vibration bands.


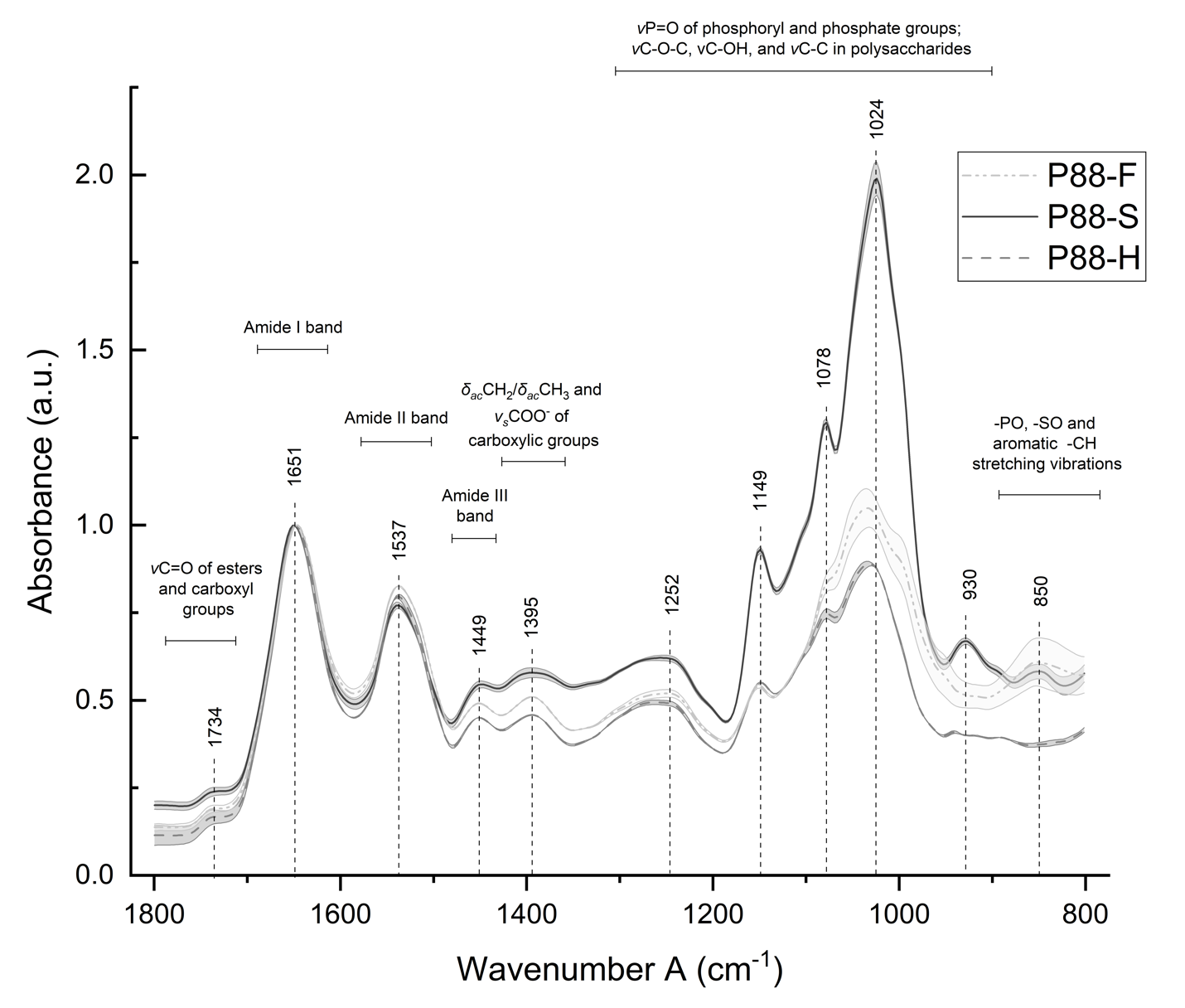
Figure S7: Averaged FTIR low-frequency fingerprint region spectra (n=3) for P88 experiments under freshwater (-F), native saline (-S), and hypersaline (-H) conditions. Key spectral regions are labelled and include ~1740 cm⁻¹ (νC=O of esters), ~1650 cm⁻¹ (amide I), ~1540 cm⁻¹ (amide II), ~1455–1300 cm⁻¹ (amide III), 1388 cm⁻¹ (*δ_ac_*CH2/*δ_ac_*CH3 and ν*_s_*COO^-^), ~1149–915 cm⁻¹ (carbohydrates and polysaccharides), ~1078–915 cm⁻¹ (phosphates and carbohydrates), and < 915 cm⁻¹ (-PO, -SO and aromatic -CH). The P88-F, P88-S, and P88-H treatments differ noticeably in the ester, phosphoryl, and carbohydrate regions.


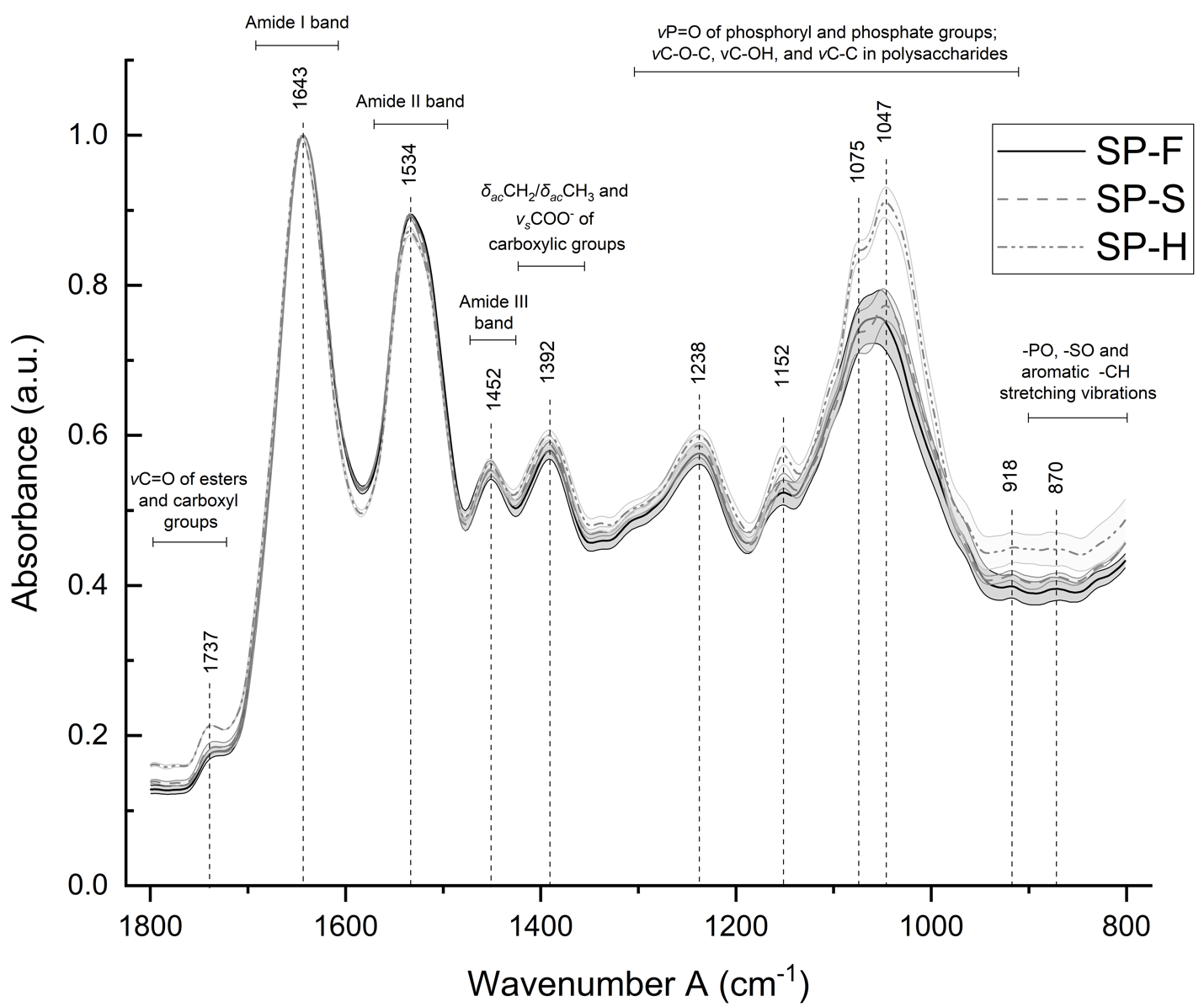


Figure S8: Averaged FTIR low-frequency fingerprint region spectra (n=3) for SP experiments under native freshwater (-F), saline (-S), and hypersaline (-H) conditions. Key spectral regions are labelled and include ~1740 cm⁻¹ (νC=O of esters), ~1650 cm⁻¹ (amide I), ~1540 cm⁻¹ (amide II), ~1455–1300 cm⁻¹ (amide III), 1388 cm⁻¹ (*δ_ac_*CH2/*δ_ac_*CH3 and ν*_s_*COO^-^), ~1149–915 cm⁻¹ (carbohydrates and polysaccharides), ~1078–915 cm⁻¹ (phosphates and carbohydrates), and < 915 cm⁻¹ (-PO, -SO and aromatic -CH). The SP-F, SP-S, and SP-H treatments differ noticeably in the ester, phosphoryl, and carbohydrate regions.

#### **Lipid Region Spectra**

Table S8: Peaks, calculated areas, and R_3/2_ values for P68, P88, and SP treatments. Larger R_3/2_ values are interpreted as less saturated, more branched lipid profiles, while lower values correspond to more saturated and less branched lipids. More branching and less saturation are consistent to more fluid membranes, while more saturation and less branching are linked to less fluid membranes.

|  | P68 | | P88 | | | SP | | |
| --- | --- | --- | --- | --- | --- | --- | --- | --- |
|  | P68-F* | P68-S | P88-F | P88-S* | P88-H | SP-F* | SP-S | SP-H |
| *v_as_*CH_3_ |  |  |  |  |  |  |  |  |
| Peak (cm^‑1^) | 2960 | 2959 | 2959 | 2959 | 2960 | 2959 | 2962 | 2971 |
| Area | 3.09 | 2.23 | 2.38 | 1.04 | 2.66 | 15.85 | 12.67 | 21.40 |
| *v_as_*CH_2_ |  |  |  |  |  |  |  |  |
| Peak (cm^-1^) | 2925 | 2924 | 2924 | 2924 | 2924 | 2919 | 2920 | 2915 |
| Area | 4.86 | 4.39 | 3.95 | 3.37 | 4.65 | 7.54 | 6.98 | 18.85 |
| R_3/2_ | 0.64 | 0.51 | 0.60 | 0.31 | 0.57 | 2.10 | 1.87 | 1.13 |


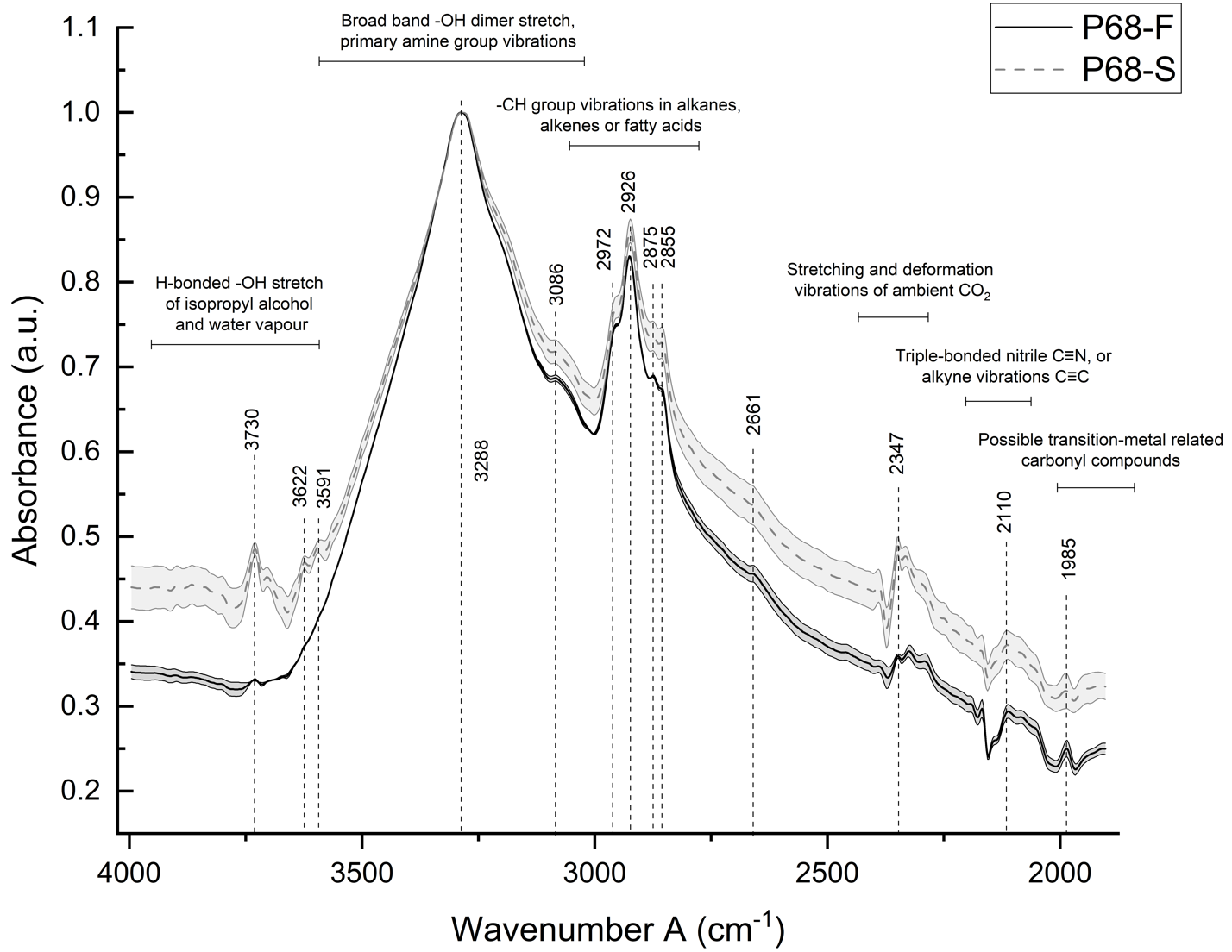


Figure S9: Averaged FTIR high-frequency lipid region spectra (n=3) P68 experiments. Regions are labeled and correspond to hydroxyl and water-related spectral vibrations, or possible triple bond and metal-related bond vibrations. Key regions of interest are the -CH group vibrations in the ~2970 – 2855 cm^-1^ range that represent vibrations of alkanes, alkenes, and fatty acids.


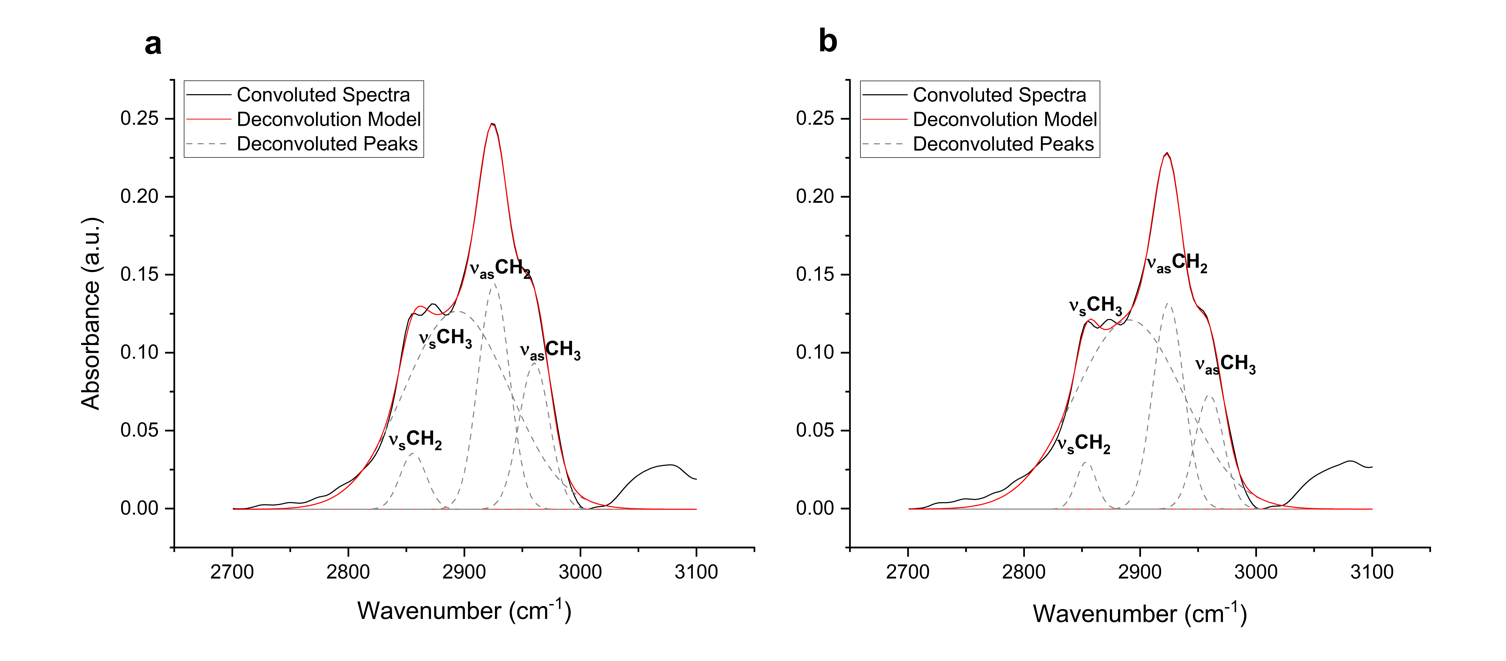
Figure S10: Spectra deconvolution for the hydrocarbon region of the (a) P68-F and (b) P68-S treatments. Deconvolution separated both the ν_as_ and ν_s_ vibration bands of methyl and methylene, of which the proportional area under the peak of ν_as_ were used to calculate the R_3/2_ ratio.


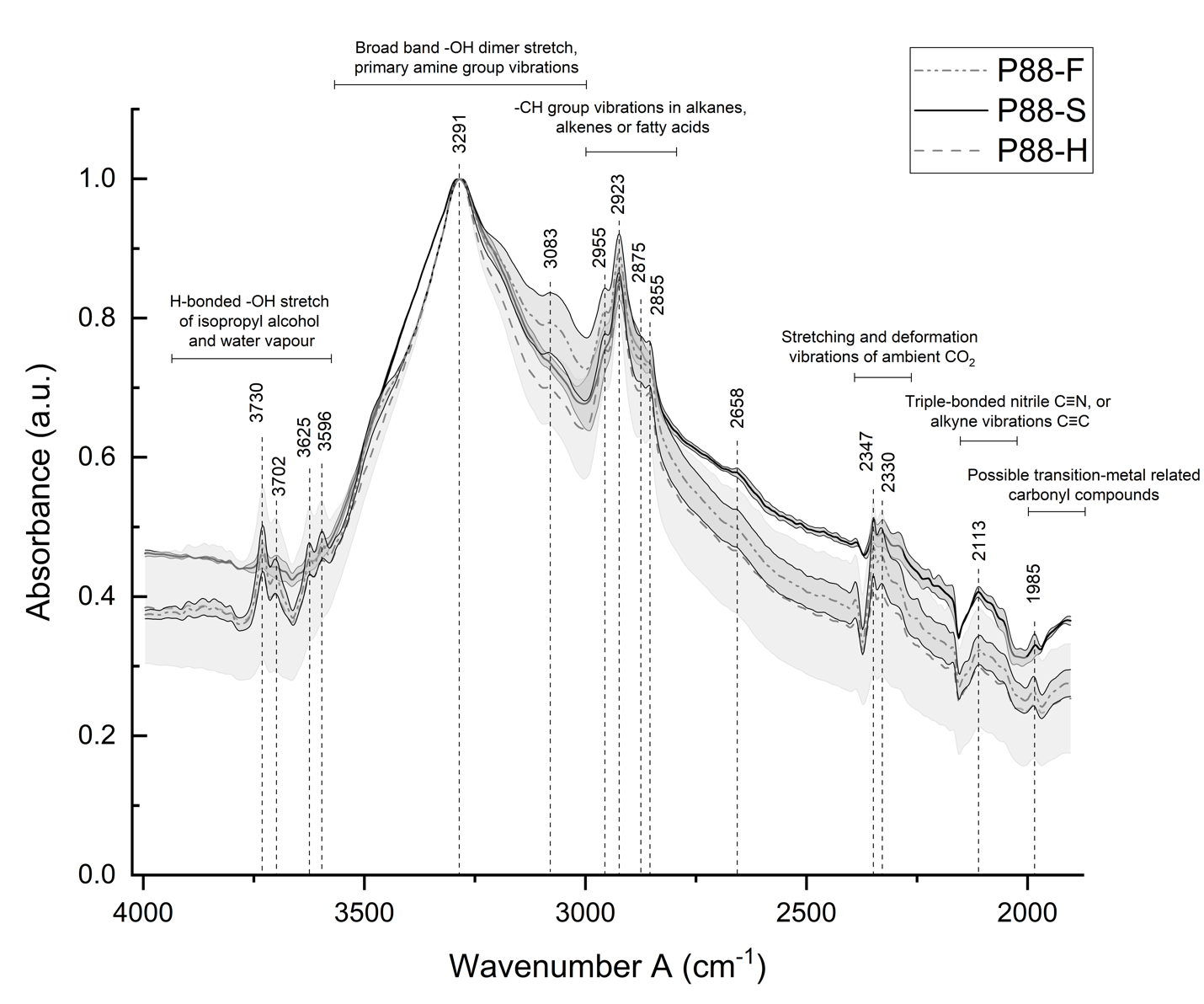
Figure S11: Averaged FTIR high-frequency lipid region spectra (n=3) for the P88 experiments. Regions are labeled and correspond to hydroxyl and water-related spectral vibrations, or possible triple bond and metal-related bond vibrations. Key regions of interest are the -CH group vibrations in the ~2970 – 2855 cm^-1^ range that represent vibrations of alkanes, alkenes, and fatty acids.


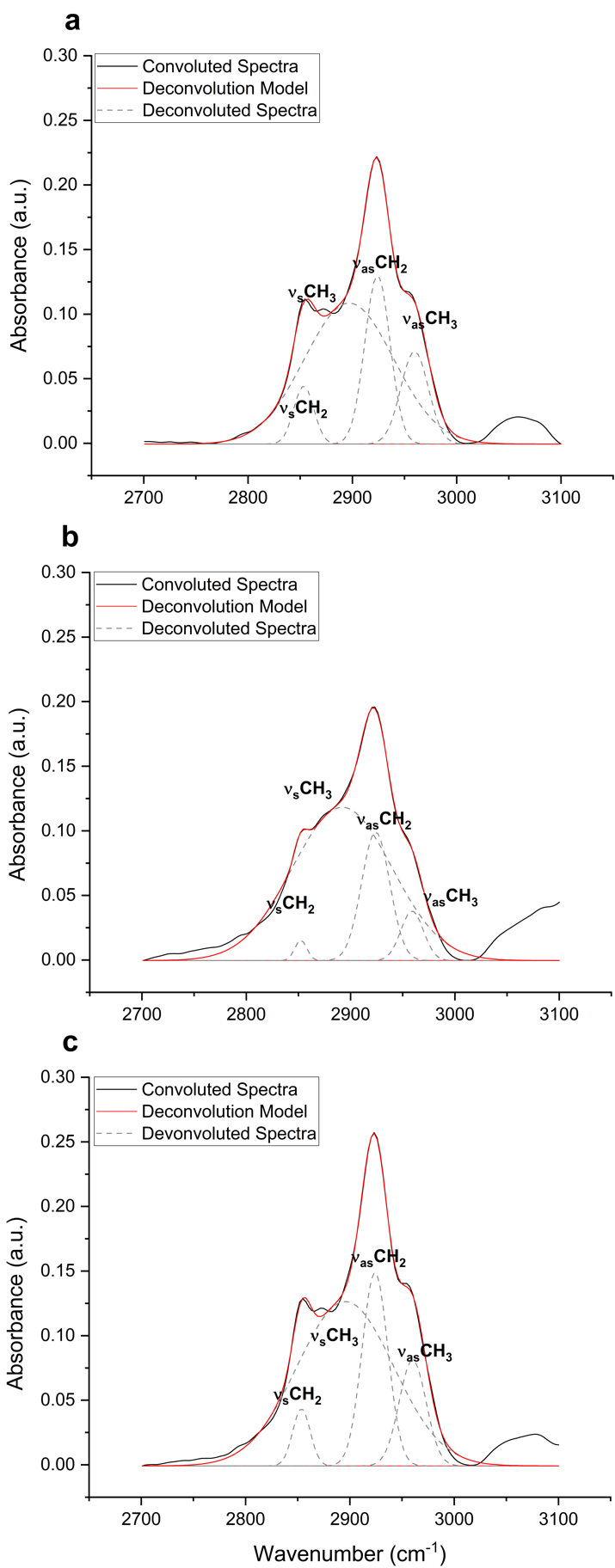


Figure S12: Spectra deconvolution for the hydrocarbon region of the (a) P88-F, (b) P88-S, and (c) P88-H treatments. Deconvolution separated both the ν_as_ and ν_s_ vibration bands of methyl and methylene, of which the proportional area under the peak of ν_as_ were used to calculate the R_3/2_ ratio.


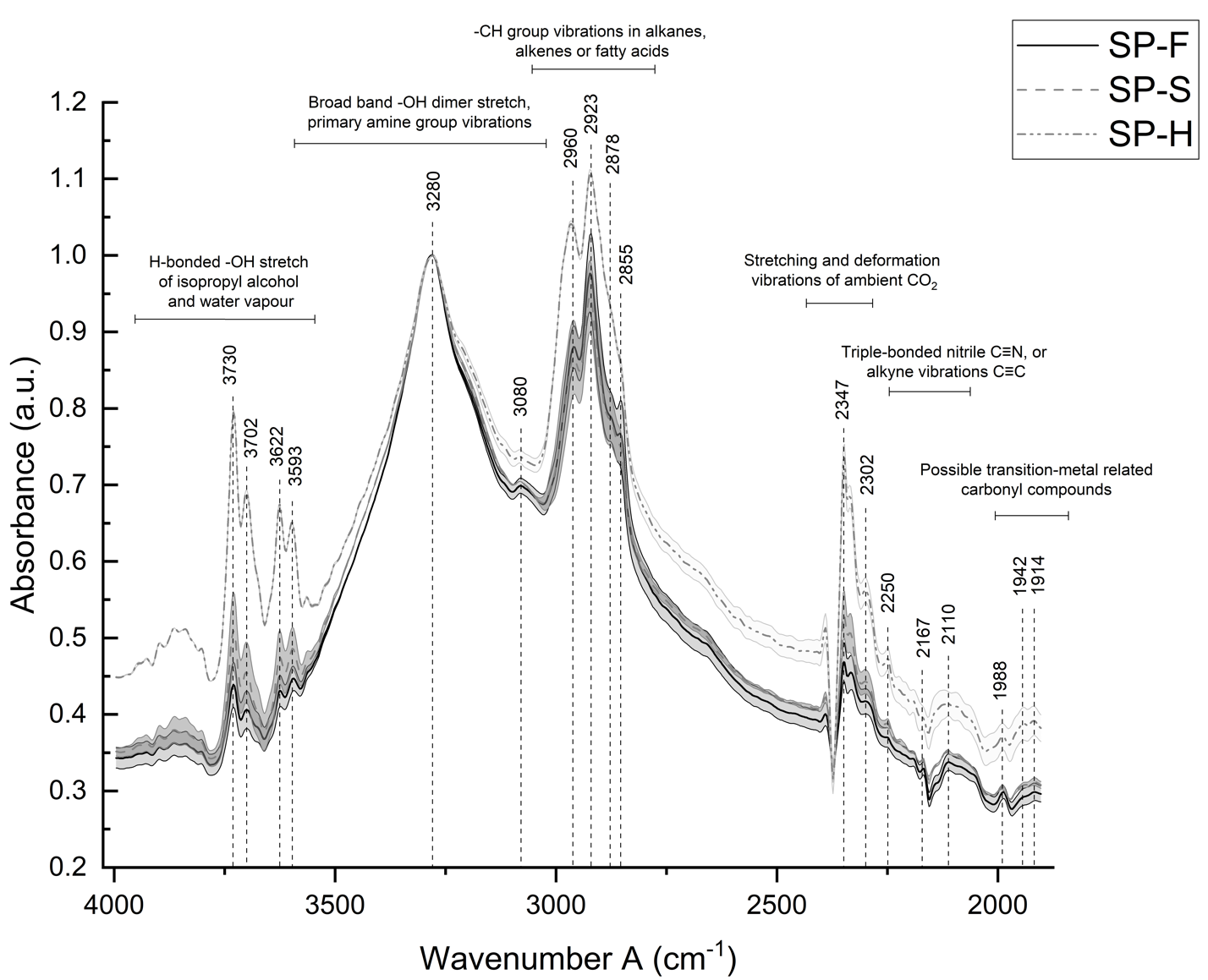


Figure S13: Averaged FTIR high-frequency lipid region spectra (n=3) for the SP experiments. Regions are labeled and correspond to hydroxyl and water-related spectral vibrations, or possible triple bond and metal-related bond vibrations. Key regions of interest are the -CH group vibrations in the ~2970 – 2855 cm^-1^ range that represent vibrations of alkanes, alkenes, and fatty acids.


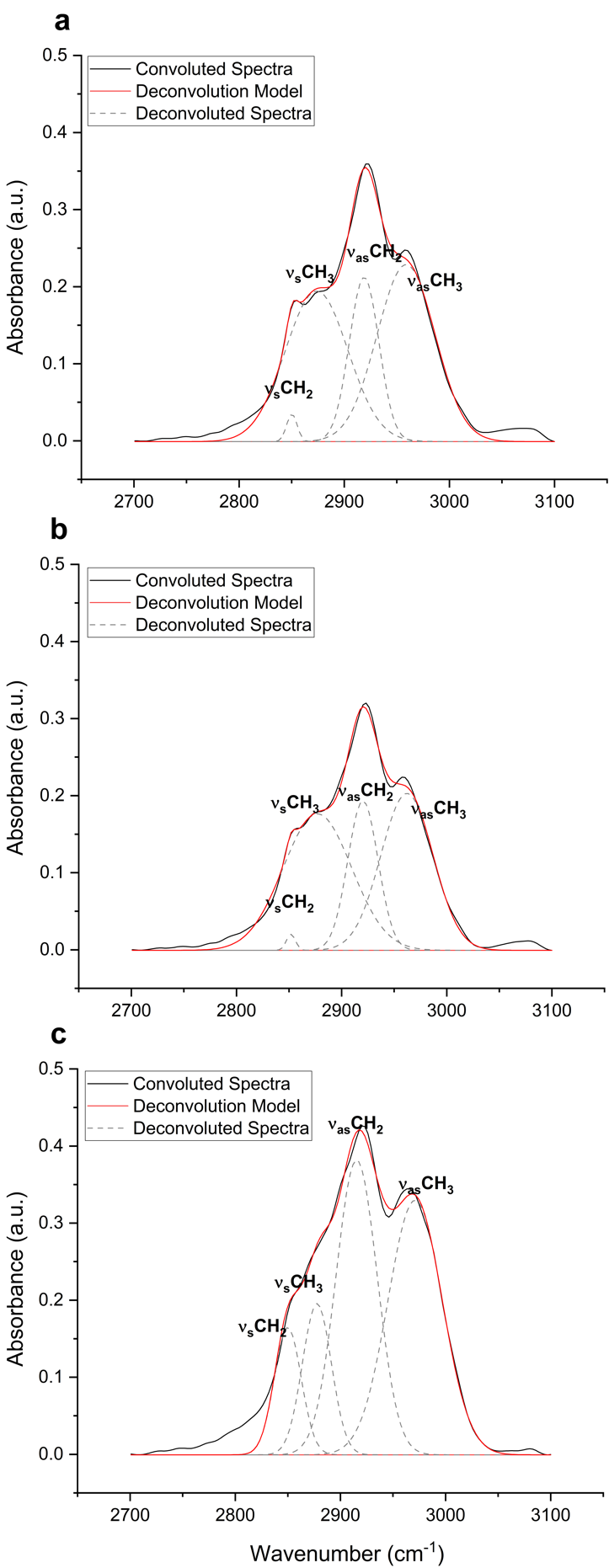


Figure S14: Spectra deconvolution for the hydrocarbon region of the (a) SP-F, (b) SP-S, and (c) SP-H treatments. Deconvolution separated both the ν_as_ and ν_s_ vibration bands of methyl and methylene, of which the proportional area under the peak of ν_as_ were used to calculate the R_3/2_ ratio.

### **XPS**

XPS calculations and deconvolution for individual peaks and overall fractions were based on methods from Paulo *et al.* (2018). The weight fraction ratios of protein (C_Pr_/C), polysaccharide (C_PS_/C), and hydrocarbon-like compounds (C_HC_/C) to total carbon were estimated according to the following equations:

$[N/C]_{obs}=0.279(C_{Pr}/C),$ (eq 1)

$[O/C]_{obs}=0.325(C_{Pr}/C)+0.833(C_{Ps}/C)$ (eq 2)

$1=(C_{Pr}/C)+(C_{Ps}/C)+(C_{HC}/C)$ (eq 3)

Solving the system of equations provided the proportion of carbon associated with each molecular constituent.

## **References**

Cox, J.S., Smith, D.S., Warren, L.A., and Ferris, F.G. (1999) Characterizing Heterogeneous Bacterial Surface Functional Groups Using Discrete Affinity Spectra for Proton Binding. *Environ Sci Technol* **33**: 4514–4521.

Diloreto, Z.A., Garg, S., Bontognali, T.R.R., and Dittrich, M. (2021) Modern dolomite formation caused by seasonal cycling of oxygenic phototrophs and anoxygenic phototrophs in a hypersaline sabkha. *Sci Rep* **11**: 4170.

DiStefano, C., Zhu, M., and Mîndrilã, D. (2009) Understanding and Using Factor Scores: Considerations for the Applied Researcher. *Practical Assessment, Research, and Evaluation* **14**:.

Dittrich, M. and Sibler, S. (2005) Cell surface groups of two picocyanobacteria strains studied by zeta potential investigations, potentiometric titration, and infrared spectroscopy. *Journal of Colloid and Interface Science* **286**: 487–495.

Dittrich, M. and Sibler, S. (2006) Influence of H ^+^ and Calcium Ions on Surface Functional Groups of *Synechococcus* PCC 7942 Cells. *Langmuir* **22**: 5435–5442.

Paulo, C., Kenney, J.P.L., Persson, P., and Dittrich, M. (2018) Effects of Phosphorus in Growth Media on Biomineralization and Cell Surface Properties of Marine Cyanobacteria Synechococcus. *Geosciences* **8**: 471.
